# Machine Learning for Designing Perovskites and Perovskite‐Inspired Solar Materials: Emerging Opportunities and Challenges

**DOI:** 10.1002/advs.74952

**Published:** 2026-03-24

**Authors:** Yangfan Zhang, Yiming Xia, Ali Shakiba, Hongrui Zhang, Xiaojing Hao, Priyank V. Kumar, Mahesh P. Suryawanshi

**Affiliations:** ^1^ School of Photovoltaic and Renewable Energy Engineering University of New South Wales Sydney New South Wales Australia; ^2^ School of Chemical Engineering University of New South Wales Sydney New South Wales Australia

**Keywords:** machine learning, perovskites, perovskite‐inspired materials, solar energy conversion

## Abstract

The development of perovskites and perovskite‐inspired materials (PIMs) is driven by the need for efficient, non‐toxic and stable solar energy conversion technologies. While halide perovskites exhibit outstanding optoelectronic properties, their practical deployment remains hindered by toxicity concerns and long‐term instability. Conventional experimental and computational approaches, though effective, are often limited by high costs and low throughput, prompting the need for data‐driven strategies. In this review, we provide a comprehensive analysis of machine learning (ML)‐driven approaches for predicting key properties such as bandgap, stability, and lattice constants in perovskite and PIMs systems. We outline a complete ML workflow, from target identification and data collection to feature engineering and model selection across supervised, unsupervised, and reinforcement learning frameworks. Special attention is given to the transferability of ML strategies developed for halide perovskites to the more chemical diverse PIMs landscape. By highlighting recent progress and current limitations, we provide a critical roadmap for integrating ML into the rational design and discovery of next‐generation non‐toxic, stable solar materials. These insights are expected to accelerate the discovery‐to‐deployment cycle for low‐toxicity, high‐efficiency solar absorbers and catalyze innovation across the broader field of data‐driven energy materials.

## Graphical abstract

This review offers a comprehensive comparison between perovskites and perovskite‐inspired materials (PIMs), focusing on their crystal structures, electronic properties, and chemical compositions. It evaluates the applicability of machine learning (ML) descriptors and models across both material classes. The review outlines recent ML advancements, details a complete ML framework for material discovery, and discusses the challenges and future prospects of applying ML to accelerate the development of high‐performance photovoltaic materials.

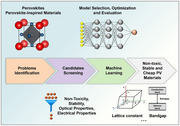

## 1 Introduction

Perovskite materials have rapidly advanced as promising photovoltaic (PV) candidates due to their unique crystal structure and excellent optoelectronic characteristics [1]. Lead (Pb)‐based halide perovskites (LHPs), in particular, have received significant attention as excellent PV absorber materials, achieving remarkable progress in power conversion efficiencies (PCEs) from 3.8% to 27% within just over a decade [2]. These materials offer efficient light absorption, high efficiency, and a simple fabrication process [3]. However, despite their significant potential, LHPs including well‐known examples such as both inorganic or hybrid organic‐inorganic perovskites (HOIPs) face critical challenges such as Pb toxicity and poor environmental stability [4, 5]. To address these issues, significant efforts have been made to find safer, lead‐free alternatives also known as perovskite‐inspired materials (PIMs). PIMs have gained significant momentum in recent years as next‐generation PV candidates, offering the potential to combine desirable optoelectronic properties with improved environmental stability and lower toxicity. These materials aim to retain the key advantages of LHPs such as tunable bandgaps, strong light absorption, and defect tolerance, while addressing critical issues such as Pb toxicity and structural instability under ambient conditions [6, 7]. The design and screening of such materials, however, remains a major challenge due to the vast and complex compositional space they occupy.

Traditional discovery of such new energy materials has relied heavily on trial‐and‐error experimentation. While density functional theory (DFT) has emerged as a particularly powerful and widely adopted tool for predicting quantum level material properties and has supported the high throughput screening of extensive compounds databases, thereby reducing reliance on purely experimental methods [8]. Although DFT is considerably faster than many‐body perturbation methods like GW, its computational costs remains significant compared to more efficient alternatives such as machine‐learned interatomic potentials (MLIPs) especially when in high‐throughput workflows involving millions of hypothetical candidates or complex chemical compositions [9, 10]. MLIPs can directly be trained on DFT reference data then reproduce potential energy surfaces with near‐DFT precision (typically within 5–10 meV atom^−^^1^ in energy and 0.05 eV Å^−^^1^ in forces) while being 10^3^–10^5^ times faster than DFT. This enables atomistic simulations involving up to millions of atoms and extended nanosecond‐to‐microsecond time scales, allowing realistic modeling of processes such as phase transitions, defect dynamics, and thermal transport [11].

Beyond atomistic potential modeling, the broader field of machine learning (ML) has also demonstrated remarkable potential in accelerating materials discovery [8, 12, 13]. ML models, when trained either on DFT‐derived data or experimental data, can rapidly and accurately predict material properties such as bandgap, formation energy, decomposition energy (E_hull_) and lattice constants at a fraction of the computational cost [14, 15, 16, 17, 18, 19]. This enables efficient screening of the vast material composition spaces associated with PIMs, allowing faster identification of non‐toxic, stable and promising candidates for solar energy conversion. While a few excellent review papers have discussed PIMs developments [20, 21], and the application of ML in halide perovskites [8, 17, 22] no comprehensive review to date has systematically started from the structural and compositional similarities between perovskites and PIMs, then exploring the extent to which ML descriptors are shared between these two material families and how ML strategies on perovskites can be applied on PIMs.

**FIGURE 1:**
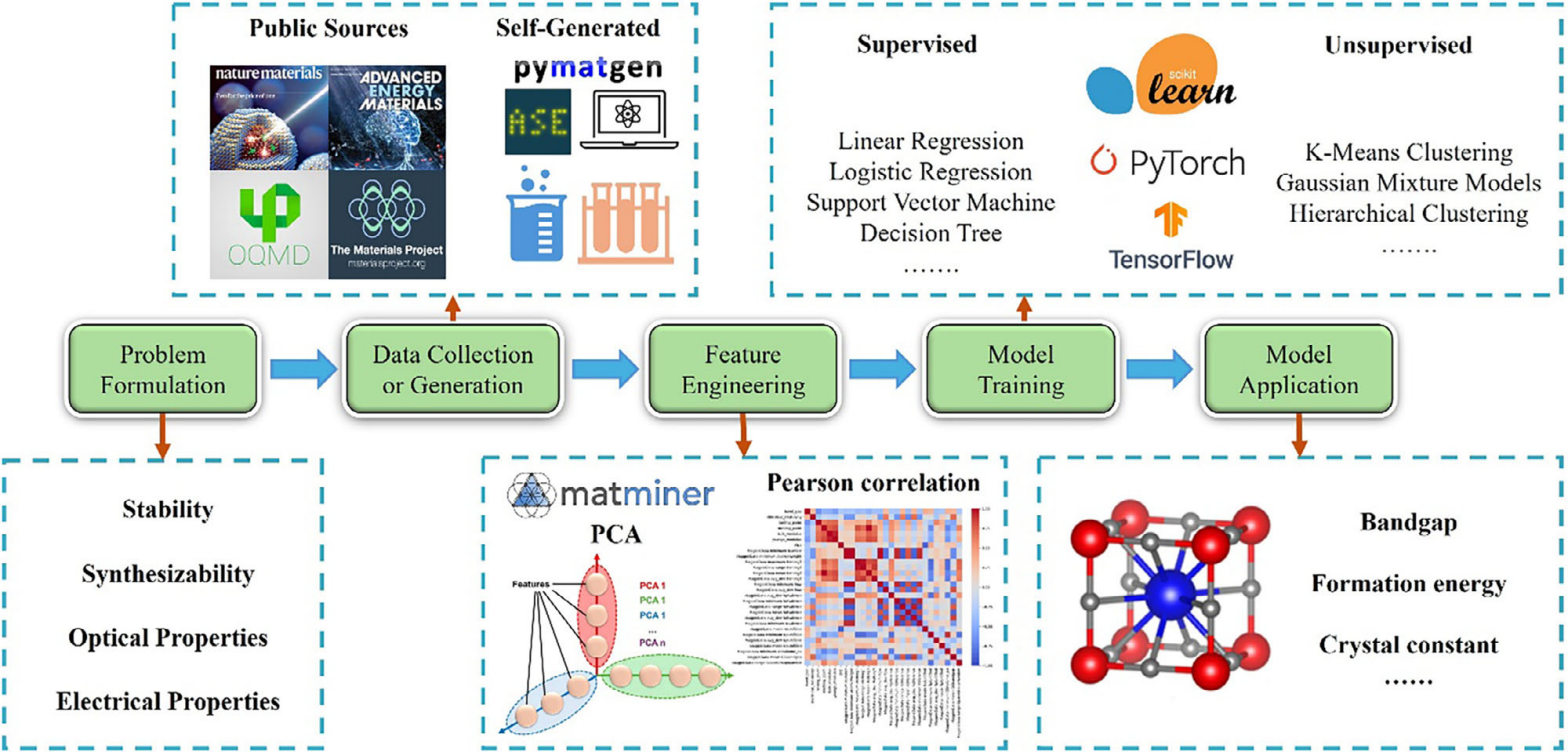
A complete workflow of an ML model. Structured by problem formulation, data collection or generation, feature engineering, model training, and application, data sources, feature engineering methods, and ML algorithms are included.

In this review, we address that gap. We provide a comprehensive analysis of ML applications in perovskites and PIMs, evaluating differences in crystal structures, electronic structures, and chemical compositions. We critically examine the generalizability of ML frameworks, from feature engineering to model selection, and highlight key advances, limitations, transfer strategies. This review is organized as follows: Section 2 presents structural, dimensional, and compositional insights into perovskites and PIMs. Sections 3, 4, 5, 6, 7, 8 outlines the complete ML workflow applied to these materials, as illustrated in Figure 1, including algorithms from foundational to state‐of‐the‐art, and their performance in terms of usage and accuracy in relevant materials systems. Sections 9 and 10 offers a recent key progress of ML applications in both perovskites and PIMs classes. Finally, Section 11 discusses open challenges such as data scarcity, model interpretability, and physics‐informed learning and proposes future directions for accelerating ML‐driven discovery of next‐generation PIMs. Given that the manuscript contains abbreviations spanning different fields, we provide a consolidated list of all abbreviations and their definitions in Table S1 to facilitate clarity and ease of reading.

## 2 Structure, Dimension, and Composition of Perovskites and PIMs

**FIGURE 2:**
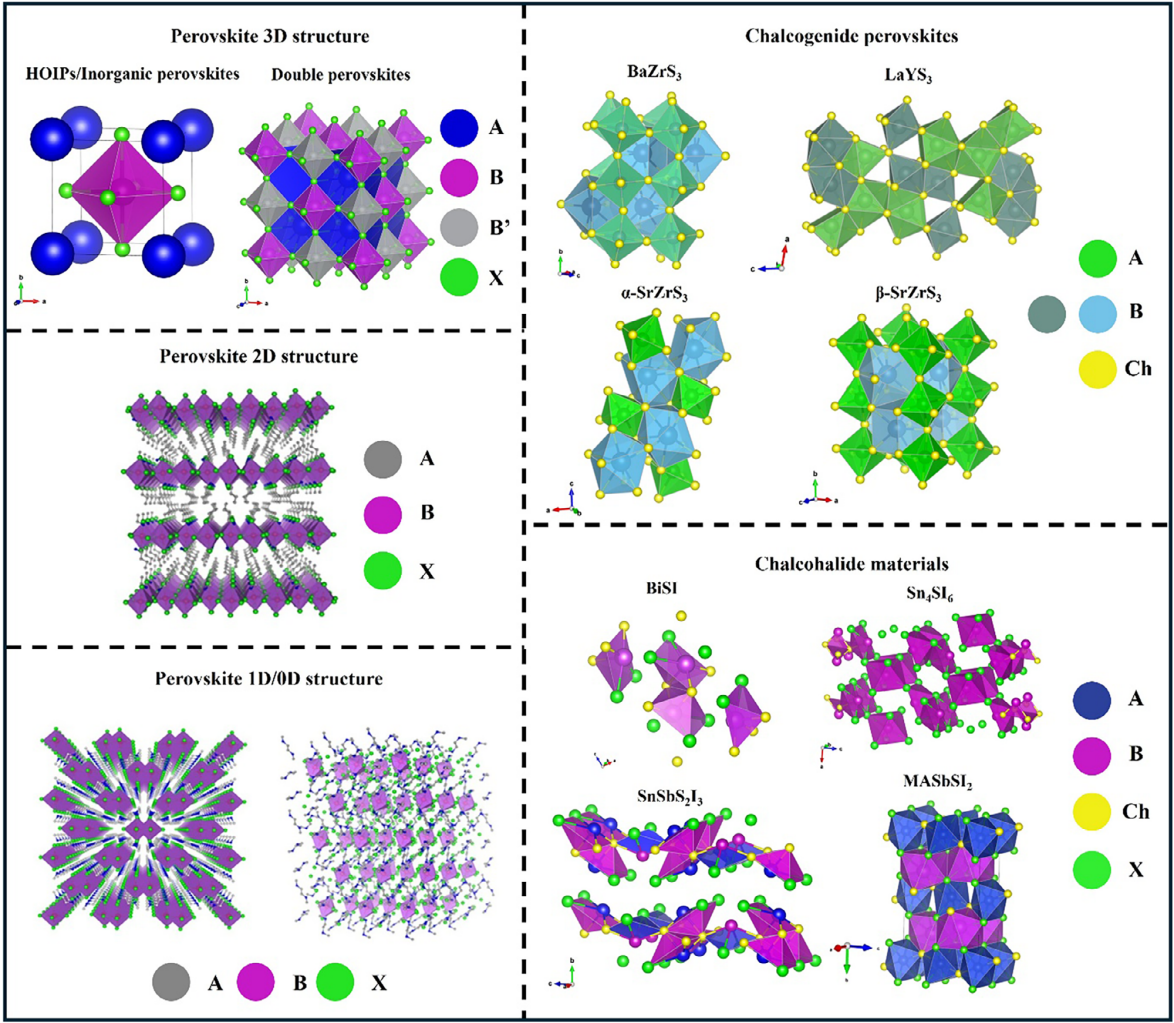
Illustration of the crystal structure of different perovskites and PIMs. Perovskite 2D, 1D, and 0D structure reproduced with permission [28]. Copyright 2018, Elsevier. Chalcogenide perovskite's structure reproduced under terms of the CC‐BY license [29]. Copyright 2021, The Authors, published by IOP Publishing.

With the development of energy material research, perovskites have demonstrated significant potential in PV, photocatalysis (PC), and other fields due to their excellent photoelectric properties, rich chemical diversity and tunable structure [23, 24, 25, 26, 27]. Building on the three‐dimensional (3D) perovskite framework, extensive efforts have led to the advancement of low‐dimensional counterparts—such as 2D, 1D, and 0D perovskites [28], which also exhibit remarkable potential for PV applications [29]. Meanwhile, PIMs further enhance the functionality and stability of perovskites by introducing structural modifications or substituting specific elements within the conventional perovskite lattice [21]. Figure 2 illustrates the schematic representations of various perovskites and PIMs, highlighting their structure, composition differences and similarities. With the widespread adoption of ML in materials science, an important research direction is the transfer of ML methodologies from perovskites to PIMs. The structural and compositional similarities between perovskites and PIMs provide a feasible basis for this transfer. Therefore, before exploring ML strategies in depth, it is essential to comprehensively analyze the structural and chemical characteristics of both for subsequent research. This chapter systematically compares the structural and chemical characteristics of perovskites and PIMs, tracing the transition from traditional 3D perovskites to low‐dimensional perovskites and exploring the development trends of PIMs. We analyze how PIMs inherit and extend key properties of perovskites and their potential applications in PV. By comparing their structural and chemical properties, we establish a theoretical foundation for the migration of ML strategies, which will be further explored in subsequent chapters.

### 2.1 Perovskites

One of the most widely studied energy materials in recent years is halide perovskite material [30]. The research of halide perovskite materials applied to PV applications started in 2009; Miyasaka and his team first reported the use of organic‐inorganic lead halide perovskite materials CH_3_NH_3_PbI_3_ (MAPbI_3_) as light absorbers in dye‐sensitized solar cells and achieved a PCE of about 3.8% [31]. In 2012, Kim and co‐workers reported the first solid‐state perovskite solar cells with a PCE of 9.7% and 500 h stability, where the devices are stored in air at room temperature without encapsulation [32]; this became a milestone of halide perovskite materials for PV applications. Afterward, research on perovskite PVs experienced remarkable and rapid growth, leading to record efficiencies reaching 27% in recent years [2], and is approaching the PCE of the most efficient crystalline silicon solar cell (27.3%) [33]. ABX_3_ is a typical structure formula for 3D perovskite, where A is a monovalent organic or inorganic cation, B is an octahedrally coordinated divalent cation with a smaller radius, and X is usually oxygen or a monovalent halide such as Cl, Br, or I [34]. Theoretically, most elements in the periodic table can be the A or B sites of ABX_3_ to form perovskites, but the typical perovskite crystal structure can only be maintained when B site cation has a +2 oxidation state and the A site ionic radius can fit within the crystal structure [29]. In details, there are two main factors usually used to judge the structure formability and phase stability of perovskite, which are Goldschmidt tolerance factor *t* [35], and octahedral factor µ [36] as can be seen in Equations (1) and (2), where *r_A_*, *r_B_*, *r_X_* are ionic radii of A, B, and X respectively.

(1) $$t=\frac{r_{A}+r_{x}}{\sqrt{2}\left(r_{B}+r_{x}\right)}\;$$

(2) $$\mu =\frac{r_{B}}{r_{x}}$$

Empirically, stable perovskite structures should have t ranging from 0.81 to 1, while a µ value between 0.44 and 0.90 [37], which can only be achieved by a limited number of compositions [38]. Meanwhile, recent studies indicate that the accuracy of Goldschmidt tolerance factor *t* is insufficient especially for halide system [39]. To address this problem, Bartel et al. [40] introduced a new tolerance factor τ, which can be represented as

(3) $$\tau =\frac{r_{x}}{r_{B}}\;-n_{A}\left(n_{A}-\;\frac{r_{A}/r_{B}}{\ln r_{A}/r_{B}}\right)$$

where, *n_A_* is the oxidation state of A. When τ < 4.18 indicates the perovskite structure. This new descriptor achieves a much higher accuracy (92%) compared to the traditional t (74%) and the prediction accuracy of halides system increased from 31% to 91%.

Currently, three main types of 3D perovskite are widely researched for PV applications: HOIPs, inorganic perovskite, and double perovskite.

#### 2.1.1 Hybrid Organic Inorganic Perovskites

In addition to the HOIPs mentioned above with organic cations at the A site, in fact, the X site of HOIPs can also be replaced from the traditional halide element to an organic anion. The introduction of these organic components gives HOIPs more functionality and structural flexibility, which is unattainable by inorganic perovskite [39]. At the same time, their diverse structures and chemical spaces provide more opportunities to tune and regulate their physical properties through simple chemical modifications [41]. Since the X site is changed to an organic anion, the tolerance factor also needs to be adjusted by the following equation. Where ***h***_***x***_ is the length of the X site molecular ions.

(4) $$t=\frac{r_{A}+r_{x}}{\sqrt{2}\left(r_{B}+h_{x}/2\right)}\;$$

**FIGURE 3:**
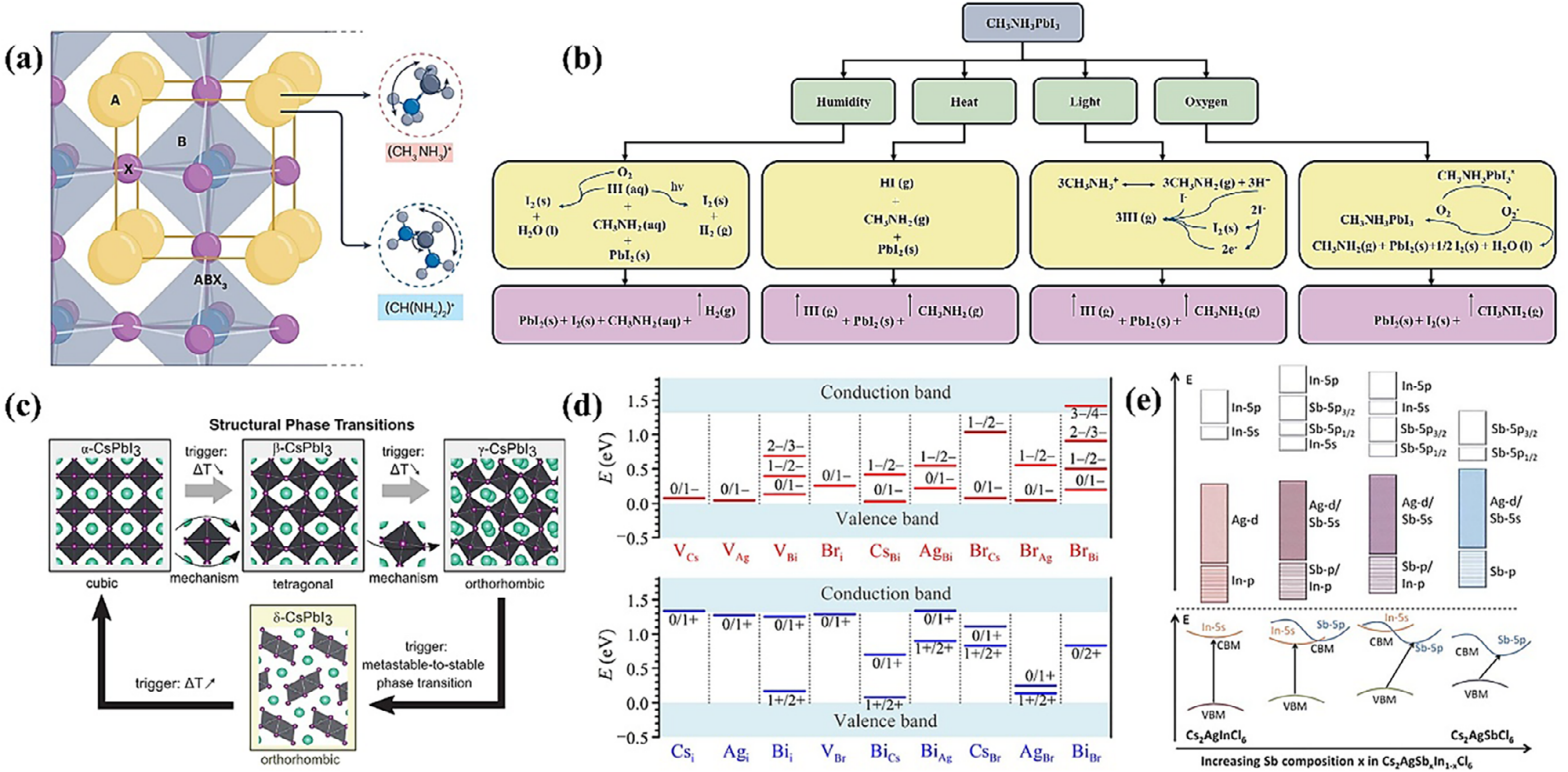
Comprehensive illustration of the main challenges of HOIPs, inorganic perovskites, and double perovskites. (a) Illustration of the dynamic movement of A site organic cation reproduced with permission [37]. Copyright 2025, Springer Nature Limited. (b) The decomposition route of HOIPs under humidity, heat, light, and oxygen reproduced with permission [45]. Copyright 2016, Angewandte Chemie International Edition. (c) Structural phase transitions of CsPbI_3_ among cubic (α), tetragonal (β), orthorhombic (γ), and metastable (δ) phases, governed by octahedral distortions and temperature triggers reproduced with permission [49]. Copyright 2019, The American Association for the Advancement of Science. (d) calculated transition energy levels e(q/q’) for intrinsic acceptors (up) and intrinsic donors (down) in Cs_2_AgBiBr_6_ reproduced with permission [50]. Copyright 2016, WILEY‐VCH GmbH & Co. KGaA, Weinheim. (e) The schematic representation of electronic structure changing of Cs_2_AgInCl_6_ by Sb doping reproduced with permission [51]. Copyright 2017, Royal Society of Chemistry.

Moreover, another key characteristic of HOIPs is the dynamic movement; the organic cations in HOIPs (occasionally also occurring on the X‐site) can undergo rotation, vibration, or multidirectional movement as can be seen in Figure 3a, leading to a disordered state, while for inorganic perovskites there are only off‐center displacement of A‐site cations [39]. When temperature decreases or environmental conditions shift, these organic molecules may become fixed in specific orientations, transitioning into an ordered state. This disorder‐to‐order transformation inevitably alters hydrogen bonding and intermolecular interactions, such as van der Waals and dispersion forces, thereby influencing the crystal symmetry of HOIPs. Symmetry changes can further lead to octahedral tilting, displacements, and order–disorder often result in more complicated phase transitions in HOIPs [42]. The interplay between the A, B, and X sites during phase transitions in HOIPs gives rise to different functionalities, such as conductivity [43] and dielectric properties [44] that are not observed in pure inorganic perovskites. Diverse A and X site options greatly expand the structural and chemical versatility of HOIPs, but the instability caused by the reaction of these molecules with the external environment prevents HOIPs from being further commercialized in the PV field [45]. Consequently, improving the stability of HOIPs stands as a primary research focus in this field. The instability of HOIPs mainly occurred under the following four environmental conditions: humidity, heat, light, and oxygen [45, 46, 47, 48]. The specific degradation mechanism under each condition is shown in shown in Figure 3b. Therefore, the general research direction to solve the stability of HOIPs in the field of materials is to screen out the combination of suitable HOIPs in the huge molecular space group so that it has the appropriate geometric structure and thermodynamic stability to obtain a more stable phase.

#### 2.1.2 Inorganic Perovskites

To overcome the inherent instability of HOIPs, inorganic perovskites are attracting more attention due to their better thermodynamic stability under high temperatures. Among them, Cs‐based inorganic perovskite shows the best potential, which can maintain the original composition and crystal structure under high‐temperature conditions up to 400°C [52]. It is also the best choice for the preparation of tandem solar cells because of the suitable wide bandgap [53]. At the same time, inorganic perovskite materials also have the excellent optoelectronic properties of HOIPs, including high carrier mobility and long carrier lifetime [54], and the PCE of recorded CsPbI_3_ has reached to 21% [55]. Although inorganic perovskites have developed rapidly, they also face the problem of further commercialization which raise from their phase stability and phase transition. For example, CsPbI_3_ exhibits four distinct phases—cubic (α), tetragonal (β), orthorhombic (γ), and non‐perovskite yellow (δ). In the stable α phase (above 350°C), the material features a perfect three‐dimensional perovskite lattice where [PbI_6_]^4^^−^ octahedra are connected by corners with a Pb─I─Pb bond angle of 180°, endowing it with excellent optoelectronic properties ideal for PV applications. When the temperature decreases, the bond angle is then reduced to approximately 170° (at around 260°C) and 150° (at around 175°C), and the structure transforms into the β and γ phases, respectively; these phases retain the three‐dimensional network, albeit with slightly distorted octahedra. In contrast, the δ phase, characterized by non‐corner‐sharing octahedra, exhibits a much larger bandgap and, consequently, poor light absorption and charge transport properties, making it unsuitable for solar cell use [49]. Notably, the phase transition process is reversible; when the temperature increases to above 350°C, the δ phase can transfer to α phase again. The phase transition route can be seen in Figure 3c. Researchers believe that there are two main reasons for the phase transition of Cs‐based inorganic perovskites: first, the tolerance factor of Cs‐based perovskites is usually close to the critical ideal value (about 0.81), which makes the material itself unstable in phase transition; second, the eccentricity of Cs^+^ ions will introduce local lattice strain, further destroying the ideal cubic symmetry. This structural mismatch makes it difficult for the lattice to maintain a stable cubic phase [52]. There are currently two research directions for solving the inorganic perovskite phase transition problem: crystal size reduction and doping. Crystal size reduction specifically refers to the use of quantum dot preparation methods, which leads to the formation of a more symmetrical crystal structure [56]. The doping technique is currently the most widely studied method. Doping new ions could modify the surface of the perovskite crystal or be incorporated into the crystal lattice, replacing one of its substituents [57]. For example, several doping strategies can be implemented to enhance the stability of Cs‐based perovskites. One approach involves A‐site doping to mitigate the inherent Cs off‐centering. Additionally, replacing the A‐site with a larger cation, performing monovalent or heterovalent doping at the B site (such as Sn^2^^+^, Ge^2^^+^) [58, 59], or incorporating smaller anions at the X‐site can collectively shift the tolerance factor away from its critical value. These modifications work together to stabilize the perovskite lattice and reduce the propensity for phase transitions [60, 61, 62]. Meanwhile, the band structure and the corresponding optical and electronic properties, such as light absorption coefficient, bandgap, and charge carrier diffusion length, can be significantly altered by element substitution on the B and/or X sites [63].

#### 2.1.3 Double Perovskites

During the search for low‐toxicity B site alternatives in perovskites, In, Sb, and Bi have emerged as promising substitutes for Pb. However, because these elements typically exhibit a +3‐oxidation state, they cannot form the conventional ABX_3_ perovskite structure. This issue is circumvented by adopting a double perovskite architecture with the formula A_2_B'B″X_6_. In this structure, the A site and X site remain occupied by monovalent cations and halides, respectively, while two Pb^2^^+^ ions are replaced by a pair of monovalent B′ and trivalent B″ cations to maintain overall charge neutrality. Consequently, the crystal is composed of alternating [B'X_6_]^5^^−^ and [B″X_6_]^3^^−^ octahedra. The availability of a wide range of A site candidates (both inorganic and organic), diverse options for B site combinations, and various halide compositions at the X site provides expanded opportunities for tailoring material properties. Therefore, extensive investigations into double perovskites are essential [64, 65, 66, 67]. Theoretically, double perovskites could combine the high performance of hybrid organic‐inorganic perovskites (HOIPs) with the enhanced stability of inorganic ABX_3_ perovskites. However, the reported properties of these materials have not yet reached ideal levels. For example, halide double perovskites such as Cs_2_AgBiBr_6_ have achieved a power conversion efficiency of only 6.37% since their initial PV application in 2017 [68, 69], despite its excellent stability which can maintain a stable phase in ambient air (with a relative humidity of ∼60%) and in the dark for 3 months [70]. The current low efficiency performance of double perovskite is caused by two main factors: large/indirect bandgap, and the lower defect tolerance compared to traditional ABX_3_ inorganic perovskite. Bandgaps of double perovskites normally range from around 2 to 3.4 eV [71], making them less ideal for single‐junction PV applications, although bandgaps around 2 eV are still suitable for use as top cells in tandem solar cells. Therefore, researchers have also tried to use doping technology to change the electronic structure of double perovskite to lower the bandgap. Tran et al. [51] demonstrated their bandgap engineering of Cs_2_Ag(Sb_x_In_1−x_)Cl_6_ by doping Sb to partially substitute the original In element. In this way, its CBM electronic structure was successfully changed as a result for smaller bandgap, and due to the impact from the Sn‐5p orbital, the bandgap type was also changed from direct to indirect as can be seen in Figure 3d. The indirect bandgap normally yields low absorption coefficients (10^2^–10^4^ cm^−^^1^ in the visible region [72]). Compared with conventional ABX_3_ perovskites, double perovskites exhibit comparatively lower defect tolerance. In detail, Xiao et al. [50] studied twenty different point defects, including vacancies, cation‐on‐anion antisites, anion‐on‐cation antisites, and interstitials for Cs_2_AgBiBr_6_. Their study showed that AgBi and BiAg antisites, Bi and Br vacancies, are not thermally ionized within the lattice as can be seen in Figure 3e, forming uncontrollable non‐radiative recombination channels (governed Shockley‐Read‐Hall recombination), which eliminate the charges and deteriorate device performance [73]. Cs_2_AgBiBr_6_ thus shows defect intolerance, possessing more deep‐level defects compared to lead‐based ABX_3_ perovskites.

#### 2.1.4 Low‐Dimensional Perovskites

**FIGURE 4:**
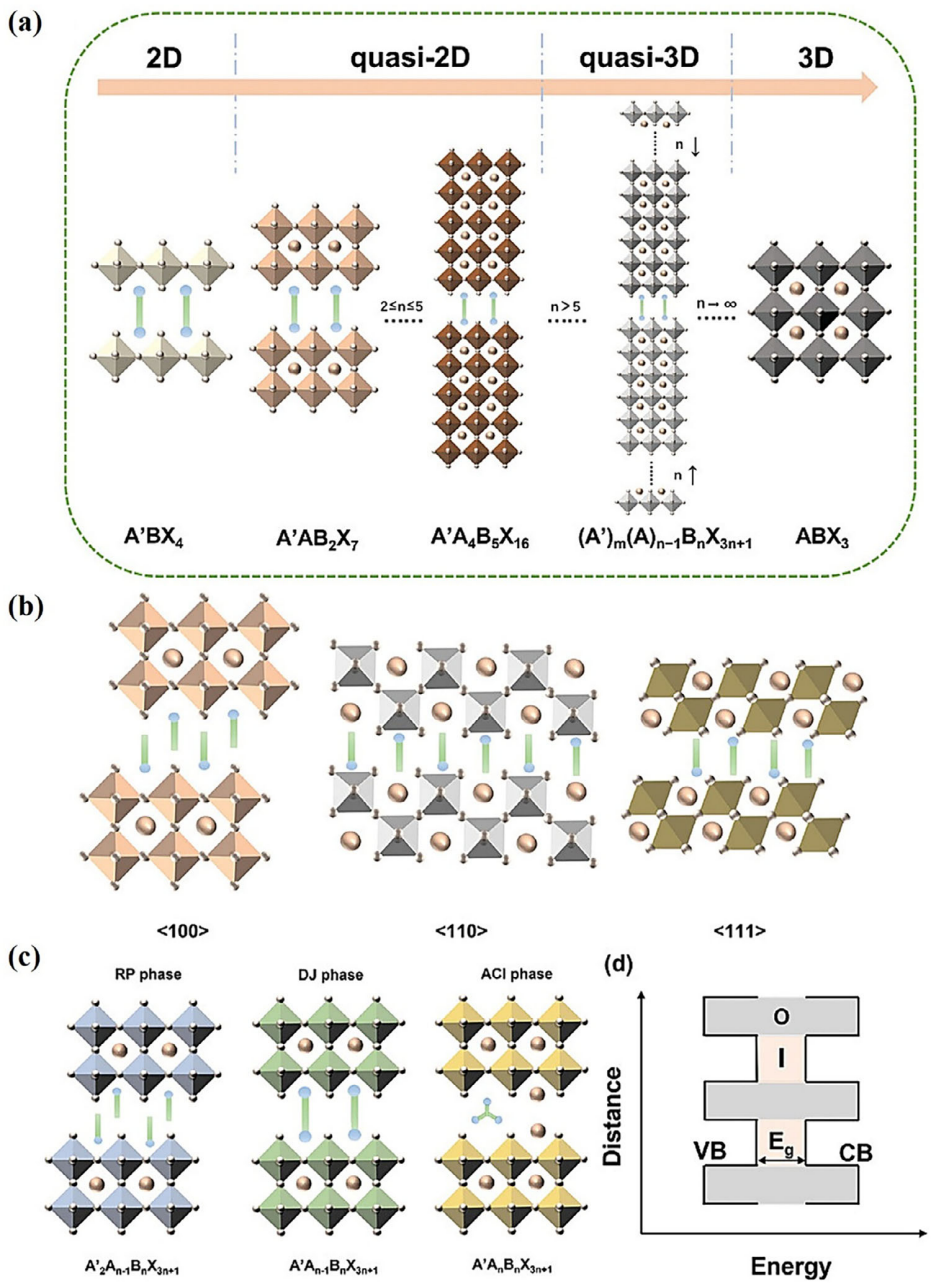
Schematic diagram of 2D perovskites reproduced with permission [76]. Copyright 2024, Wiley‐VCH GmbH. (a) different n‐values. (b) different crystal plane orientations. (c) 2D perovskite structure of RP phase, DJ phase, and ACI phase. (d) Schematic diagram of a quantum well (QW), where “O” and “I” represent organic and inorganic layers in 2D perovskites.

The earliest studies on 2D metal halide perovskites were conducted by Maruyama et al. in 1986 [74]. Compared with the narrow compositional range of 3D metal halide perovskites, which are limited by satisfying the t‐factor constraint, the introduction of larger organic A′‐site cations (organic spacers) can relax the constraints on the formation of viable 2D perovskites [75]. 2D perovskites are composed of two parts: an organic interlayer and an inorganic octahedral layer. The introduction of interlayer cations not only introduces a new compositional dimension, represented by A′, but also brings unprecedented structural complexity, thus achieving tunability of optoelectronic properties [76]. The dimension reformation process can be seen in Figure 4a. Normally, the chemical formula of a 2D perovskite can be written as (A’)_m_(A)_n–1_B_n_X_(3n+1)_, where the m represents the number of interlayer cations (usually 1 or 2) and n represents the number of inorganic layers [77], where the value of n can be controlled by adjusting the stoichiometric ratio of the A’‐site cations to the A‐site cations. When the thickness (number of layers) of the inorganic layer increases, the bandgap and exciton binding energy (Eb) of the material decreases [78]. However, when n is equal to 1, it is generally considered to be a pure 2D perovskite, in which the exciton binding energy is too high and therefore unsuitable for PV applications. When 2 ≤ n ≤ 5, it is called a quasi‐2D perovskite; when n > 5, it is called a quasi‐3D perovskite. When n tends to infinity, its dimension can be considered to be restored to a 3D perovskite. 2D perovskites can be divided into layered perovskites with <100>, <110>, and <111> orientations (see Figure 4b). Among them, the <100> orientation is dominant. The introduction of most interlayer cations will lead to a <100> oriented structure, making it the most common 2D perovskite structure. According to the charge of the cations, the interlayer cations can be divided into monovalent (+1) and divalent (+2). The different geometries of these cations have different effects on the crystal structure of 2D perovskites, because they may form ionic bonds with a single inorganic layer or two inorganic layers, respectively, spanning the organic spacer layer. Therefore, based on the <100> orientation, 2D perovskites can be further divided into three types according to the molecular structure of the organic cations: Ruddlesden–Popper (RP) phase, Dion–Jacobson (DJ) phase, and alternating cation interlayer (ACI) phase [79], as can be seen in Figure 4c. In most cases, the RP phase corresponds to monovalent cations, while the DJ phase corresponds to divalent cations. However, there are special cases where monovalent cations present in the DJ phase and divalent cations present in the RP phase [80, 81]. These exceptional cases, along with the ACI phase [82, 83, 84] involve very complex organic content, so we will not discuss it in more depth here. Currently, RP phase is the most widely studied one, characterized by the diversity of interlayer cations, which enables constructing a vast perovskite network. The 2D RP phase successfully improved the stability of the Sn and Ge based perovskite especially in terms of anti‐oxidation performance, when a larger organic cations butylammonium (BA) and 2‐phenylethylammonium (PEA) are incorporated with Sn or Ge in a halide system, it forms (PEA)_2_GeI_4_ and (PEA)_2_SnI_4_ with direct bandgap 2.12 and 2.2 eV respectively [85]. It showed better stability in humid environments [86]. In addition, this type of compound itself can also maintain thermal stability under high temperature conditions above 200°C [87]. DJ phase also shows great performance in terms of stability. For example, the (4AMP)(FA)_3_Sn_4_I_13_ absorber formed using 4‐(aminomethyl) piperidinium as the organic component only lost 9% of its initial PCE (initial efficiency of 4.22%) after continuous operation in a N_2_ atmosphere [88]. Even better results were achieved in a system using 1,4‐butanediamine (BEA)—(BEA)FA_2_Sn_3_I_10_, which maintained 90% of its initial efficiency of 6.43% after 1000 h of operation under similar conditions and also performed well after 200 h of operation at room temperature [89]. However, compared with 3D perovskites, 2D perovskites show poor charge transport properties due to the anisotropy of the inorganic skeleton. There are many reasons for this phenomenon. First, due to the structural symmetry, the dispersion of the inorganic layer in the vertical direction is almost zero in 2D perovskite materials, indicating weak interlayer coupling [90]. The movement of electrons in the direction perpendicular to the perovskite crystal plane is restricted, resulting in localized and narrowed energy bands. In addition, the distortion of the electronic structure caused by the electron‐phonon interaction in the Jahn‐Teller effect further narrows the localized energy band, resulting in a wider bandgap for 2D perovskites than their 3D counterparts [91]. Second, the unique physicochemical properties of the organic spacer cation ligands have a significant impact on the optoelectronic properties of 2D perovskites. The hydrophobic organic spacer cations can separate the conductive inorganic layers to form a quantum well structure as can be seen in Figure 4d. In this structure, the inorganic layer and the organic spacer layer act as potential wells and barriers, respectively. Organic interlayers destroy the orbital hybridization between adjacent inorganic layers, thereby confining photogenerated charge carriers within the inorganic layers, severely hindering external charge transport [92]. In addition, in 2D perovskites, the dielectric constant of organic interlayer cations is much lower than that of inorganic octahedral layers, which leads to the dielectric solid confinement effect. The charge screening ability provided by organic interlayers is weak, thereby enhancing the Coulomb interaction between photogenerated electrons and holes. The significant quantum confinement effect and the dielectric confinement effect together contribute to the higher exciton binding energy in 2D perovskites, making it easier for excitons to form rather than dissociate into free electrons and holes, thereby limiting the charge transport capacity and reducing the PCE [79, 93]. Third, the introduction of organic interlayer cations changes the stacking mode of inorganic octahedral layers and distorts the bond angle and bond length between metals and halogens. These changes significantly affect the orbital overlap between metal and halogen ions, thereby affecting the bandgap [76]. Fourth, 2D perovskite films prepared by solution method are usually composed of multiple quantum wells, whose width (n value) and orientation are randomly distributed, which will lead to reduced mobility and diffusion length of charge carriers from the perspective of the overall material [94, 95]. Although 2D perovskites exhibit good stability, low charge transport is still a big challenge for PV applications. Generally, strategies to improve the performance of 2D perovskite are still to adjust the A', B and X site components. For the A site, Hautzinger et al. [96] studied a variety of A‐site cations and found that FA^+^ and MA^+^ are “ideal” choices. Cations that are too large (such as EA^+^, DMA^+^) or too small (such as Cs^+^) can cause distortion of the inorganic layer, increase defects and non‐radiative recombination, and increase the bandgap. At the same time, Zhou et al. [97] found that FA^+^ helps reduce non‐radiative recombination centers and form high‐quality, highly oriented films. So, there is no need to engineer the A site at current stage. At A’ site, By introducing functional groups (such as ‐F, ‐OH, ‐CN) or heteroatoms (such as S), the distance and electronic coupling between inorganic layers can be finely controlled, which helps to improve charge transport and reduce trap density [98]. The selection of aromatic or conjugated organic cations can reduce the dielectric mismatch between the organic and inorganic layers, reduce the exciton binding energy, and enhance the crystal stability through π‐π interactions [99, 100]. Using symmetrical imidazolium‐based cation such as benzimidazolium (Bn) and benzodiimidazolium (Bdi) can narrow the bandgap. These cations have shown evidence that effectively narrow down the bandgap of Sn Based halide system to 1.81 and 1.79 eV for Bn_2_SnI_4_ and BdiSnI_4_, respectively [85]. At B site, where is usually Pb^2^^+^ (or Sn^2^^+^, Bi^3^^+^ and other alternative ions). By replacing or partially doping, the band structure, carrier mobility and defect tolerance of the material can be adjusted [101]. At X site, Halogens play a vital role in two‐dimensional perovskites, and their influence can be summarized into three aspects: optical properties, structural regulation, and charge transport. Br^−^ and I^−^ can significantly improve the light absorption coefficient and material stability, but the diffusion rate is slow, while Cl^−^ exhibits excellent charge transport characteristics but weak absorption capacity. The different halogen ion radius, polarizability, and electronegativity will change the tolerance factor of the inorganic framework, leading to tetrahedral distortion, thereby regulating the lattice parameters, interlayer distance, and bandgap size; for example, the increase in Cl content will aggravate the lattice distortion and increase the bandgap, while affecting the formation of self‐trapped excitons and changing the photoluminescence properties [102]. In addition, halogens also change the valence band structure by regulating the degree of orbital mixing between metals and halogens, so that the bandgap gradually decreases when the I^−^ content increases [103]. Therefore, the ratio of different X site components has become an important design strategy for optimising the optoelectronic properties of 2D perovskites.

### 2.2 Perovskite‐Inspired Materials (PIMs)

PIMs are another class of semiconducting materials designed to retain the outstanding optoelectronic properties of lead halide perovskites while mitigating their instability and toxicity [29]. Most PIMs are lead‐free systems that emulate the light absorption and charge transport properties of traditional perovskites by precisely tailoring their crystal structure and chemical composition [104, 105, 106, 107].

#### 2.2.1 Chalcogenide Perovskites

**FIGURE 5:**
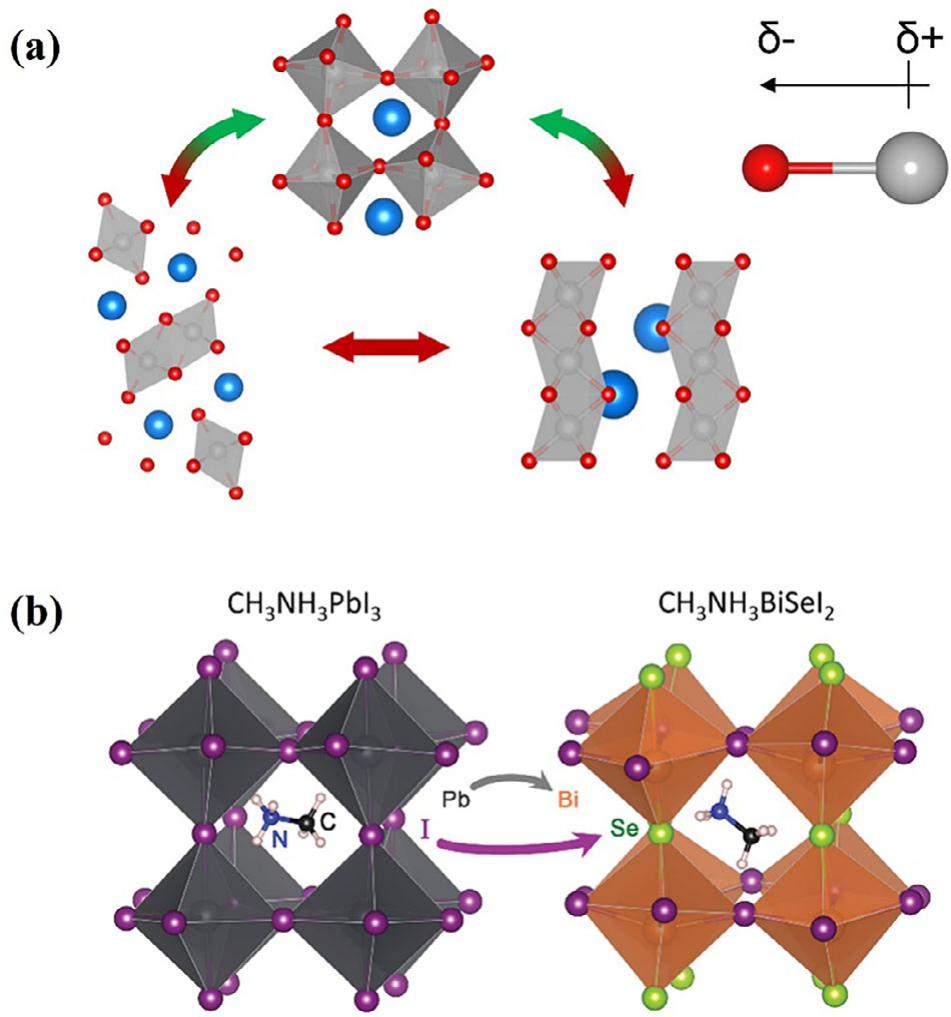
Illustration of chalcogenide perovskite structures and composition engineering of chalcohalide. (a) Distorted structures of chalcogenide perovskites reproduced with permission [111]. Copyright 2022, American Chemical Society. (b) Split‐anion approach of chalcohalide material to replace Pb in CH_3_NH_3_PbI_3_ reproduced with permission [120]. Copyright 2016, Royal Society of Chemistry.

Chalcogenide perovskite is new emerging PIMs which also have the ABX_3_ chemical formula, the A site remains occupied by monovalent or divalent cations while the B site is typically composed of high‐valent metals such as Zr^4^^+^ or Bi^3+^ and the halogen element (such as Cl, Br, I) of traditional perovskites with chalcogenide (such as S, Se, Te). Widely studied chalcogenide perovskites including BaZrS_3_, SrZrS_3_, and LaYS_3_ [29]. The composition substitution not only eliminates toxic Pb but also strengthens the metal–chalcogen bonds, thereby enhancing the thermal and moisture stability relative to traditional halide perovskites [108]. For example, the LaYS_3_ thin film perovskite has already been used in n‐i‐p cell structure, verifying the device feasibility of chalcogenide perovskite [109]. At the same time, the 1.8 eV bandgap of BaZrS_3_ is very suitable as the upper layer of silicon cells. Researchers estimate that if BaZrS_3_ correlated with silicon as a tandem solar cell, the PCE is hopefully to achieve around 35% [109]. However, this promising approach comes with its own set of challenges. The strong covalent character of the chalcogenide bonds often induces structural distortions as can be seen in Figure 5a, resulting in an orthorhombically or even hexagonally distorted perovskite lattice rather than the ideal cubic symmetry found in typical ABX_3_ perovskites [35, 110, 111]. Consequently, most chalcogenide perovskites such as BaZrS_3_ generally exhibit wider bandgaps, typically in the range of 1.7 to 2.1 eV, which can be used in tandem solar cells, but is not able to be used directly in single‐junction cells [109]. Although some elements can be used to dope anions, for example, replacing part of sulfur with a certain proportion of Se can adjust the bandgap range of BaZr(S,Se)_3_ from 1.5–1.9 eV, its structural stability will gradually decrease with the increase of Se content [112]. Therefore, optimising the bandgap by regulating the chemical composition while maintaining structural stability is a major research direction of chalcogenide perovskite.

#### 2.2.2 Chalcohalide Materials

Chalcohalide materials, which contain compounds of both chalcogen anions and halogen anions and is generally described by the general formula MChX (M is a metal cation, Ch is a chalcogen anion, and X is a halogen anion). This type of material can be considered as a derivative PIM from typical perovskite or chalcogenide perovskite, a “split anion” system formed by partially replacing halogen with chalcogen elements (or vice versa). Chalcohalide materials have shown considerable promise in various energy applications, ranging from PVs to photocatalysis and thermoelectrics. Their ability to combine the properties of both chalcogenide perovskites and typical halide perovskite allows for a versatile manipulation of their band structures and electronic behaviors, making them highly adaptable for different energy‐related technologies [113, 114]. Currently, the chalcohalide materials can be classified into four categories: heavy pnictogen chalcohalides, transition/post‐transition chalcohalides, mixed‐metals chalcohalides, and hybrid organic‐inorganic chalcohalides.

In terms of the heavy pnictogen chalcohalides, SbSI and BiSI are two examples that have been studied extensively in the early stage. They exhibit low effective mass, high absorption coefficient and suitable bandgap. In addition, since Sb^3^^+^ and Bi^3^^+^ has ns^2^ lone electron pairs, its chemical properties can mimic Pb^2^^+^ to a certain extent, thus giving the material better defect tolerance and optoelectronic properties. This type of material is not only suitable for PV applications but also shows potential advantages in fields such as ferroelectricity and photocatalysis [115, 116, 117]. For transition/post‐transition chalcohalides, this type usually uses transition metals or post‐transition metals as the M position, and its electronic structure and crystal chemistry are different from those of heavy group chalcohalides. First report about transition/post‐transition chalcohalides was in 1960 for Ag_3_SI and Ag_3_SBr, which chemical structure is also called anti‐perovskite structure [118]. Ag_3_SX (X = I, Br, or Cl) normally have a bandgap at around 0.9–1.1 eV at room temperature and is suitable for single‐junction and tandem solar cells but is currently limited by expensive and time‐consuming synthesis and deposition processes (e.g., laser ablation requires pre‐synthesis under high temperature vacuum conditions) [119]. By introducing two or more different metal ions at the M position, it forms the mixed‐metals chalcohalides that the bandgap, band edge and defect energy level can be finely controlled. The mixed metal system can not only adjust the light absorption characteristics of the material to a certain extent but also improve its crystal stability and carrier transport performance. This multi‐metal cation strategy is similar as the double perovskite for designing materials to achieve both high PCE and good stability [113]. In the hybrid organic‐inorganic chalcohalides, organic cations are combined with inorganic chalcohalide structures to form a hybrid structure similar to traditional HOIPs [120] as can be seen in Figure 5b. The hybrid structure helps to further reduce the bandgap of the material, improve interface bonding, and regulate the growth dynamics of the film. It may also introduce flexibility and adjustability unique to organic components. But at the same time, the hybrid system also faces challenges in terms of long‐term environmental stability from moisture and heat [121]. It is worth noting that for any type of chalcohalide materials, although the introduction of mixed anions can increase the covalency of the B─X bond and thus improve the structural stability, it also increases the thermodynamic difficulty of forming the perovskite phase [20]. Therefore, the research direction of chalcohalide should be to screen candidates with a suitable bandgap of thermodynamic temperature in a huge space combination group.

## 3 Candidates Screening and Problem Formulation

Whether for perovskite or PIMs, the biggest challenge lies in how to identify potential candidate materials from the nearly infinite chemical space. Currently, the most widely used method is to set up a reasonable “funnel” screening channel [29]. Specifically, limiting element selection to non‐toxic elements is an effective first step in PIMs screening, followed by further screening, including charge neutrality, valency and electronegativity can effectively reduce the search space by two orders of magnitude from 10^12^ possibilities [122]. The Goldschmidt tolerance factor *t*, octahedral factor µ and new tolerance factor τ mentioned above are the primary standard for screening traditional 3D perovskite structure candidates. Successfully applying ML to the design of perovskites and PIMs requires correctly formulating the problem into a suitable method for ML. This step shapes the project framework, influencing algorithm selection, data collection, and evaluation methods [8]. According to the discussion in the previous chapter, we can find that the main research direction of perovskite and PIMs with organic composition is the selection or modification of organic cations to solve the stability problem, and the directions of inorganic perovskites and PIMs can be summarized as composition engineering to improve photoelectric performance, and the main direction of low‐dimensional perovskite is to improve passivation capability or surface optimization capability when cooperating with 3D perovskite. So based on different demands, the screening criteria are further determined by the following four properties: stability, synthesisability, optical properties and electrical properties.

### 3.1 Stability

Thermodynamic stability is a fundamental property that determines whether a material can remain in its desired phase without decomposing into other phases under equilibrium conditions. For PV materials, achieving high thermodynamic stability is crucial to ensure durability and consistent performance over extended periods. Thermodynamic stability is typically evaluated using two key parameters: the formation energy (*E_f_*) and the energy above the convex hull (*E_hull_*). Formation energy measures the energy required to form a compound from its constituent elements. A lower *E_f_* indicates that the material is energetically favourable and less likely to decompose. Mathematically, it is expressed as:

(5) $$E_{f}=E_{total}\;-\;\sum_{i}n_{i}\mu_{i}$$

Where *E_total_* is the total energy of the compound, *n_i_* is the number of atoms of element i, and µ_*i*_ is the chemical potential of element i. For a PV material, a negative and low *E_f_* relative to competitive phases is desirable to ensure phase stability during operation. In high‐throughput computational screening and ML‐guided materials discovery, *E_hull_* is widely recognised as a more rigorous indicator for thermodynamic stability of a material than E_f_ alone. E_hull_ quantifies the energy above convex hull, the thermodynamic ground state of all competing phases at a given composition. A value of *E_hull_* =  0 indicates that the compound lies on the convex hull and is thus thermodynamically stable. Materials with *E_hull_* > 0 are metastable, meaning they may decompose into more stable phases [123]. However, metastable compounds can still be experimentally synthesizable, especially if kinetic barriers prevent decomposition. A widely adopted threshold for synthesizability is *E_hull_* ≤ 50 meV/atom, under which materials are generally considered stable enough for practical synthesis and applications [124].

### 3.2 Synthesizability

While thermodynamic and kinetic stability are foundational, they are insufficient to guarantee lab‐scale synthesizability. In many cases, compounds predicted to be thermodynamically stable remain unsynthesized due to precursor incompatibility, or complex reaction pathways [125, 126, 127, 128, 129]. To address this gap, data‐driven approaches have recently been developed to directly assess synthesizability based on historical experiment examined datasets. For example, positive‐unlabelled (PU) learning models, label experimentally confirmed entries from the Inorganic Crystal Structure Database (ICSD) as positive samples (P), while unverified computational structures from the Materials Project (MP) are treated as unlabelled data (U). Since explicit negative examples are unavailable, a subset of U is randomly selected and temporarily assigned as negative samples. Then, using both P and this sampled subset, the model is trained as a binary classifier. This process is carried out multiple times using bootstrap aggregation. The model produces a Crystal‐Likeness Score (CLscore) between 0 and 1, which shows how likely it is that the synthesis will be successful. This partially supervised strategy lets the model make statistical predictions about the structural patterns that make up synthesizable materials without needing negative data that has already been set up which have achieved predication accuracies of 75–88% [130]. Based on PU learning, Gleaves et al. [131] developed semi‐supervised teacher–student neural network which introduces a dual‐network architecture to dynamically leverage both labelled (from ICSD and PU learning) and unlabelled data (from MP). In this framework, the teacher model generates pseudo‐labels for unlabelled samples, while the student model learns from these labels and provides feedback to refine the teacher's predictions. This adaptive learning mechanism effectively mitigates bias from limited labelled data and achieves superior synthesizability prediction performance up to 92.9%, surpassing conventional PU learning approaches. Moreover, recent Crystal Synthesis Large Language Models (CSLLM) converts crystallographic data (CIF and POSCAR) into “Material Strings” that encode lattice parameters, space groups, and atomic coordinates in a textual format suitable for natural language processing. which can not only predict the synthesizability of materials with 98.6% accuracy, but also can provide precursors path for screened materials [132]. These emerging alternative approaches dramatically reduce the reliance on extensive thermodynamic and AIMD calculations, thereby accelerating the pace of materials discovery.

### 3.3 Optical Properties

The bandgap is of paramount importance among all optical properties. Only materials with appropriate bandgap types (direct / indirect) and values can serve as potential candidates for ideal PV materials and the suitability of the bandgap value is determined by Shockley–Queisser (SQ) limit [133]. According to the SQ limit, the maximum efficiency achievable for a single‐junction solar cell is approximately 33% [134]. This maximum efficiency is being continuously approached by materials with bandgap values in the range of 1.1–1.45 eV. This range includes many technologically significant semiconductors, such as crystalline silicon (Si), gallium arsenide (GaAs), and copper indium gallium selenide (Cu(In,Ga)Se_2_) across a wide range of indium‐to‐gallium ratios, as well as cadmium telluride (CdTe). Currently, the highest certified efficiency record for single junction solar cell is around 29.1%, achieved using GaAs thin film technology by Alta Devices [135, 136]. For multi‐junction or tandem solar cells, the design aims to combine semiconductor with various bandgaps to achieve higher efficiencies. An ideal configuration would involve pairing the bandgap of silicon or Cu(In,Ga)Se_2_ at approximately bandgap around 1.1 eV with a higher bandgap material in the range around 1.7 eV [137]. Therefore, the bandgap of the ideal PV material needs to meet the criteria for 1.1–1.45 eV for single‐junction or around 1.7 eV for multi‐junction (Perovskite/silicon tandem solar cells). In addition to the bandgap, the optical absorption coefficient is another key optical property. An ideal PV material should have a high absorption coefficient to absorb sunlight efficiently. This means that the material should have strong absorption capabilities in the visible and near‐infrared spectrum. Generally, materials with an optical absorption coefficient above 10^4^ cm^−1^ can be considered to have good light absorption capabilities [138]. A higher light absorption coefficient means that a thinner absorber layer can absorb most of the incident light, which is very important for reducing material costs and improving device efficiency [139]. Additionally, exciton binding energy is another important optical property worth considering. A low exciton binding energy is preferred, as it facilitates the dissociation of photo‐generated excitons (electron‐hole pairs) into free carriers, thereby improving charge collection efficiency and overall device performance [140].

### 3.4 Electrical Properties

Electrical properties are another critical factor in determining the suitability of a material for PV applications. These properties primarily influence the collection and transport efficiency of photogenerated charge carriers, directly affecting the overall PCE of solar cells. Among the key electrical properties, carrier mobility (µ) and carrier lifetime (τ) are of paramount importance [137]. Carrier mobility quantifies how quickly charge carriers (electrons and holes) can move through a material under the influence of an electric field. High carrier mobility ensures efficient charge transport to the electrodes before recombination occurs, minimizing energy losses during the process. For an ideal PV material, the carrier mobility should be sufficiently high to support long‐distance transport of charge carriers without significant scattering or recombination. However, direct prediction of carrier mobility is time‐consuming. Therefore, the effective mass can normally be used as alternative metrics to evaluate if a material has high carrier mobility. Carrier mobility is inversely proportional to the effective mass, meaning that the lower the effective mass, the higher the mobility. For example, gallium arsenide (GaAs) has an effective mass of 0.067 and a high mobility of 8500 cm^2^/V·s, while silicon (Si) has an effective mass of 1.09 and a lower mobility of 1400 cm^2^/V·s. Therefore, any compound with an effective mass below 1 is highly likely to be a promising PV material [141]. Likewise, carrier lifetime defines the average time photogenerated charge carriers (electrons and holes) exist before recombination occurs. It directly affects the efficiency of charge collection and transport within solar cells [142]. However, due to the complex dependencies, it is difficult to be captured directly with ML models, so it is also necessary to select simple and alternative targets and criteria. For example, Debye temperatures can be used. When it is greater than 500k, it can be beneficial for suppression of nonradiative combination and thus improve carrier lifetime [143]. In addition, defect tolerance has become increasingly crucial for perovskites and PIMs. Shallow energy level defects have little effect on carrier transport, while deep energy level defects will become non‐radiative recombination centers, seriously reducing carrier lifetime and device efficiency [144]. Therefore, accurately predicting defect energy levels is essential for evaluating the electronic properties of materials [145].

## 4 Data Collection and Processing

**FIGURE 6:**
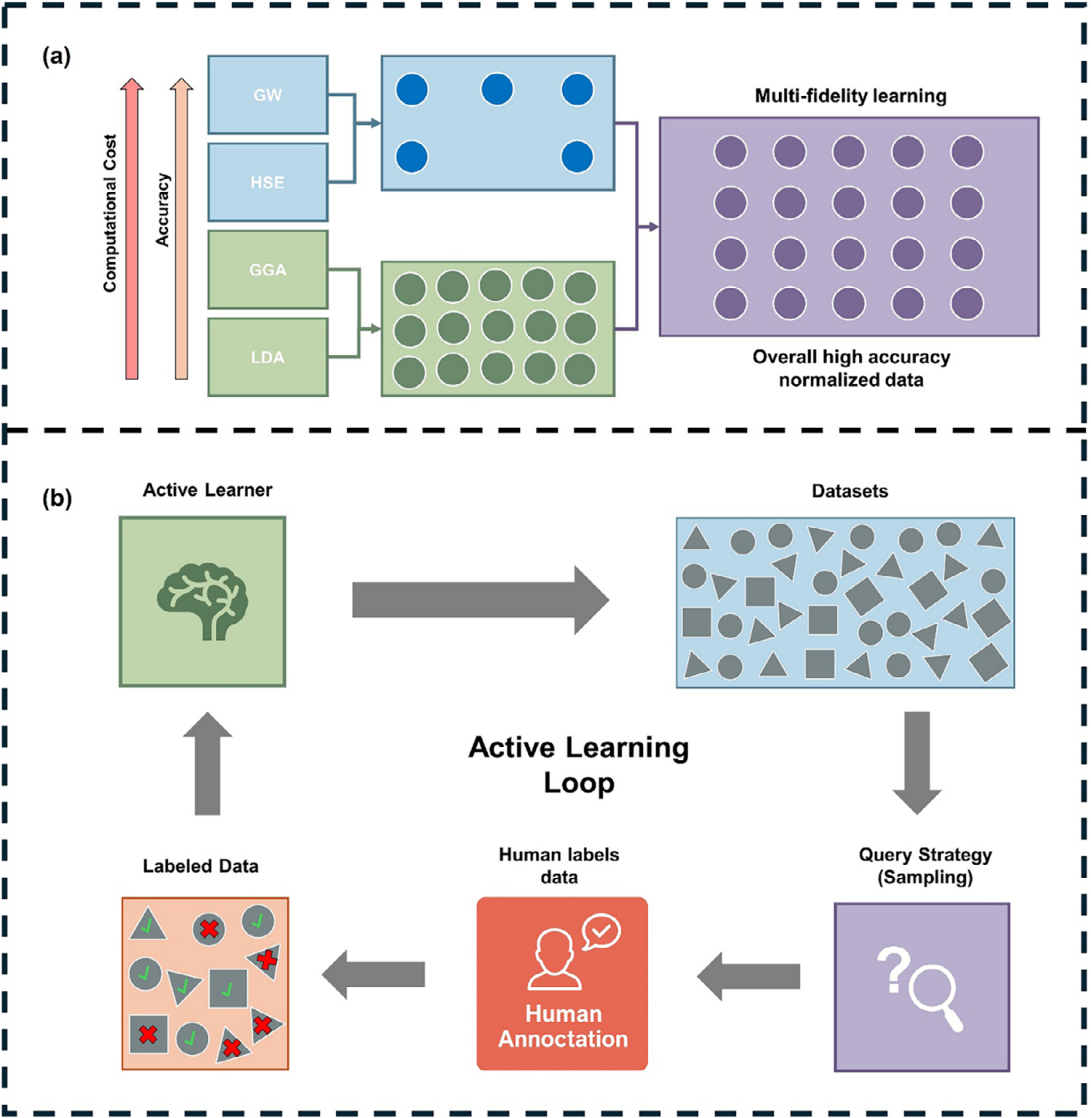
Illustration of data processing methods. (a) Multi‐fidelity learning. (b) Active learning.

The dataset utilized for ML often consists of both features (independent variables) and targets (dependent variables) that are related to the materials. Independent variables, also called features or descriptors, are the specific details that represent the structure and characteristics of materials. These details include the chemical composition, atomic or molecular parameters, structural parameters, and the technological conditions used in the synthesis process. The dependent variables are the specific properties of the materials that are influenced by the independent factors, which are also referred to as the target variables [146, 147]. For accurate ML prediction, the training dataset must be both sufficiently large and of high quality. While the required data volume varies depending on the model type and task complexity, large models such as neural networks and deep learning architectures, typically demand dataset ranging from 10^4^ to 10^6^ entries to ensure robust and generalizable performance [148]. In terms of data quality, the use of high‐quality data can prevent the consideration of erroneous, missing, or redundant information; hence we need to ensure data comes from reliable sources [8]. Current main data sources can be broadly categorized into two types: public sources and self‐generated data. Public sources are further divided into two categories: scientific literature and public databases. Scientific literature offers the latest research finding and serves as a rich resource for data, including experimental results, synthesis procedures, and property measurements [149, 150]. With the development of materials science, public databases are now becoming the main data sources in ML projects [151, 152]. Currently, there are many reliable public databases such as MP, ICSD, Open Quantum Materials Database (OQMD), etc [153, 154, 155, 156, 157, 158, 159, 160, 161, 162, 163, 164, 165, 166, 167, 168, 169, 170, 171]. These databases illustrated in Table 1 collectively store a vast array of structures and properties of materials. Self‐generated data sources can also be divided into two parts, experimental datasets and computational datasets [172]. Experimental datasets are fundamental as they offer ground truth data against which ML models can be trained and validated, it normally compiled from laboratory experiments and provide accurate data on various compounds including chemical composition, synthesis methods, crystal structures, and measured properties like bandgap, stability, carrier mobility and efficiency [173]. Computational datasets are primarily derived from DFT calculations [174, 175]. It allows researchers to simulate material properties, predict their stability, and optimize compositions for specific applications. DFT is an ab initio computational method that minimizes computational intensity of electronic structure calculations by leveraging charge density as a key variable as opposed to the more complex wavefunction. Although DFT formulation is exact, practical DFT employs approximate so‐called exchange‐correlation functionals including local density approximation and generalized gradient approximation, and also hybrid functionals, to achieve a balance between computational efficiency and accuracy for predicting electronic structures, bandgaps, phonon properties, and reaction energetics of materials [176]. Another crucial consideration in data collection is bandgap predication, the inconsistencies arising from different levels of theory in DFT calculations. For example, bandgaps computed using the GGA, such as Perdew‐Burke‐Ernzerhof (PBE) functional, are known to systematically underestimate experimental values. In contrast Heyd‐Scuseria‐Ernzerhof (HSE) estimates bandgap that are typically in much closer agreement with experimental results [177, 178, 179]. Mixing bandgap values obtained from different functionals within the same training dataset can introduce significant biases and degrade model performance. Therefore, to ensure accuracy and generalizability in ML models, it is essential to clearly distinguish and consistently use data generated from same level of theory during dataset assembly. Beyond electronic structure calculations, AIMD simulations are increasingly used to study the finite‐temperature mechanical and thermodynamic behavior of materials at the atomic scale. These simulations capture how materials respond to varying temperatures, pressures, and external forces, generating extensive time‐series data. This data is frequently leveraged in ML frameworks to develop interatomic potentials and predict transport properties such as diffusion and mechanical response [152]. Building upon these computational approaches, high‐throughput computational screening has become a powerful and systematic approach for accelerating materials discovery by automating large‐scale first‐principles calculations. It leverages computational frameworks, databases, and workflow automation tools to efficiently explore vast compositional and structural spaces, significantly reducing the time and effort required to identify promising materials for specific applications. Commonly used tools for high throughput screening can be seen in Table S2 [180, 181, 182, 183, 184, 185, 186, 187, 188]. However, due to experimental conditions, measurement technology, human bias, and other factors, the same type of experimental data may differ across different sources, leading to data inconsistency problems. To address this issue, it is essential to compare data from multiple databases and apply techniques such as data fusion [189], data reconciliation [190], and consensus methods [191]. Beyond these methods, Multi‐fidelity learning has recently become another powerful tool to combine different datasets with different levels of accuracy and computational cost. In materials science, this method allows for the integration of low‐fidelity data (such as PBE‐based DFT calculations) with high‐fidelity results (like HSE or experimental measurements) to create hierarchical models that reflect both general patterns and specific physical relationships [192]. It can move knowledge from one dataset to another by understanding how deviation are related. This makes predictions more accurate and cuts down on the need for expensive high‐level calculations [193]. This method is quite useful for perovskite and PIMs, where there aren't many experimental data and the accuracy of theoretical datasets varies depending on the functional or computational scheme used. Figure 6a schematically illustrates the fundamental principle of multi‐fidelity learning.

**TABLE 1:** Public material databases.

Database	Data Type	URL	Free	API
AFLOWLIB	Inorganic & Computational	http://aflowlib.org	Yes	Yes
ASM	Inorganic	http://www.asminternational.org	No	No
AiiDA	Alloy Phase Diagram	http://www.aiida.net	Yes	Yes
C2DB	2D	https://cmr.fysik.dtu.dk/c2db/c2db.html	Yes	Yes
CMR	Multiple (3D, 2D)	https://cmr.fysik.dtu.dk	Yes	Yes
COD	Multiple & Experimental	http://crystallography.net	Yes	Yes
Cambridge Structural Database (CSD)	Multiple	https://www.ccdc.cam.ac.uk	No	Yes
ChEMBL	Bioactive molecules	https://www.ebi.ac.uk/chembl	Yes	Yes
ChemSpider	Multiple	https://www.chemspider.com	Yes	Yes
Citrination	Multiple	https://citrination.com	No	Yes
Clean Energy Project	Solar cell	https://cepdb.molecularspace.org	Yes	No
EELS Data Base	Spectra	https://eelsdb.eu	Yes	No
GDB	Small organic molecules	http://gdb.unibe.ch	Yes	No
HTEM	Inorganic	https://htem.nrel.gov/	Yes	No
ICSD	Inorganic & Experimental	https://icsd.fiz‐karlsruhe.de	No	Yes
JARVIS‐DFT	2D	https://www.ctcms.nist.gov/~knc6/JVASP.html	Yes	No
LPF	Multiple	https://paulingfile.com	Yes	No
MPDS	Multiple	https://mpds.io/#/modal/menu	Yes	No
MatNavi	Multiple	https://mits.nims.go.jp	Yes	No
MatWeb	Engineering	http://matweb.com	Yes	No
Materials Cloud	Multiple (3D, 2D)	https://www.materialscloud.org	Yes	No
Materials Commons	Computational	http://materialscommons.org	Yes	No
Materials Project	Multiple	https://materialsproject.org	Yes	Yes
NOMAD	Multiple	https://nomad‐repository.eu	Yes	Yes
NREL Materials	Computational & Renewable	https://materials.nrel.gov	Yes	No
Nano‐HUB	Nanomaterials	http://nanohub.org	Yes	No
OMDB‐GAP1	Organic crystals	https://omdb.mathub.io/dataset	Yes	No
OQMD	Multiple & Computational	http://oqmd.org	Yes	No
PCD	Multiple	http://www.crystalimpact.com/pcd	No	No
Springer Materials	Multiple	https://materials.springer.com	No	No
Supercon	Superconducting	https://supercon.nims.go.jp	Yes	No
TEDesignLab	Thermoelectric	http://www.tedesignlab.org	Yes	No
XAFS database	Spectra	https://www.cat.hokudai.ac.jp/catdb/	Yes	No

As a complement to multi‐fidelity learning, active learning provides a dynamic strategy to further optimize data acquisition and model training. Instead of processing data in a static and linear manner on fixed dataset, active learning continuously evaluates the data within a closed loop [194, 195]. As shown in Figure 6b, the workflow begins with an initial model trained on a small labelled datasets, which then evaluates a large unlabelled dataset. A query strategy based on uncertainty, diversity, or expected model improvement, selects a subset of the most informative samples for further evaluation. These selected samples are then labelled through human annotation, and the updated labelled dataset is used to retrain the model. This creates a closed feedback loop that keeps improving predictive accuracy and generalization.

The integration of these data processing techniques into the research of perovskites and PIMs will definitely solve the data shortage problem, thereby accelerate the pace of material discovery and design.

## 5 Feature Engineering and Selection

**FIGURE 7:**
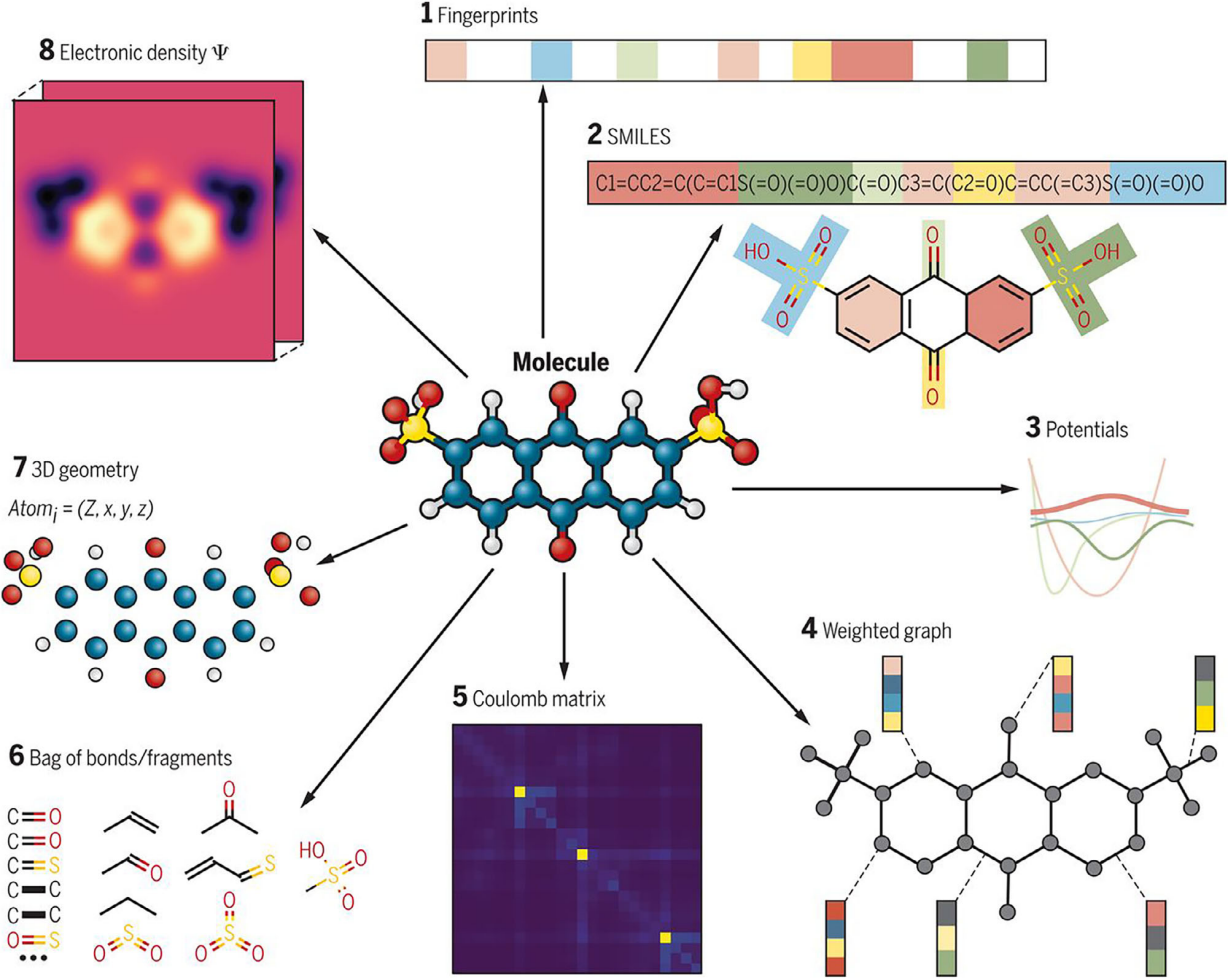
Different molecular representations commonly used in feature extraction for organic compounds in ML: (1) Molecular fingerprints — binary encodings indicating the presence or absence of specific chemical substructures; (2) SMILES strings — textual representations that describe molecular structure; (3) Potential energy surfaces — representations of molecular energetic landscapes; (4) Weighted molecular graphs — nodes (atoms) and edges (bonds) capturing topological and bonding information; (5) Coulomb matrices — numerical encodings of pairwise electrostatic interactions; (6) Bag‐of‐bonds or fragment descriptors — simplified vectorized groups reflecting local chemical environments; (7) 3D geometries — explicit spatial coordinates of atoms; and (8) Electron density maps — spatial distributions derived from quantum chemical calculations. Reproduced with permission [200]. Copyright 2018, The American Association for the Advancement of Science.

In ML, feature engineering and selection are two important steps following data collection. The primary objectives of these two processes are multifaceted: enhancing the performance of ML models, reducing computational complexity, and improving model interpretability. A complete feature engineering process should include feature extraction, preprocessing, normalization, and standardization [196]. In the context of periodic inorganic materials such as inorganic perovskites or PIMs, feature extraction is often carried out using domain‐specific tools such as Matminer [197], Pymatgen [186], the smooth overlap of atomic positions [198], and component‐based feature vector [199]. These tools can effectively generate features based on crystal structures or chemical formulas making them suitable for inorganic compounds with periodic lattices. For organic compounds, feature extraction commonly employs specialized molecular representation methods, as illustrated in Figure 7. These molecular representations are crucial for ML applications [200]. Typical representations for organic molecules include molecular fingerprints, simplified molecular‐input line‐entry systems (SMILES), potentials, weighted graph representations, Coulomb matrices, bag‐of‐bonds or fragments, three‐dimensional (3D) geometry, and electronic density distributions. Molecular fingerprints represent molecules as binary vectors indicating the presence or absence of particular chemical substructures, which allows efficient analysis of structure‐property relationships. SMILES provides a textual encoding of molecular structures, facilitating its use in cheminformatics and molecular design workflows. Potentials describe the energetic landscapes of molecules, useful in predicting their stability and reactivity. Weighted graph representations interpret molecules as graphs composed of atoms (nodes) and chemical bonds (edges), with detailed atomic and bonding information, thus enabling graph neural networks (GNNs) to effectively capture the topological and chemical characteristics. The Coulomb matrix encodes molecules based on atomic charges and spatial positions, effectively capturing internal electrostatic interactions. The bag‐of‐bonds or fragments approach decomposes molecules into distinct bonding environments or functional groups, offering intuitive and interpretable chemical descriptors. Additionally, the accurate representation of molecular geometry via 3D coordinates provides essential spatial structural information, which is crucial for predicting properties influenced by stereochemistry and molecular conformations. Finally, electronic density distributions derived from quantum chemical calculations directly reflect electron distributions within molecules, enabling precise predictions of electronic properties and chemical behavior. Utilizing these diverse molecular representation techniques significantly enhances ML models' ability to predict various properties of organic compounds, such as electronic, optical, thermal characteristics, chemical reactivity, and stability, thus supporting efficient inverse molecular design processes.

These standardized feature generation tools form the cornerstone for transferring ML strategies developed for perovskites to PIMs. By providing a unified and physically consistent feature representation framework, they bridge the structural and compositional similarities between the two material families. Once the reliability of these descriptors and corresponding ML models has been verified on large, data‐rich perovskite datasets, the entire feature–ML framework can be confidently extended to data‐augmented PIMs datasets, enabling comparable predictive accuracy and interpretability.

When data is abundant but contains redundancy and irrelevant features, feature selection becomes a crucial step in data preprocessing. It effectively addresses the ‘curse of dimensionality’, a phenomenon where the feature space becomes exponentially large and the model's performance starts to deteriorate rather than improve [201]. Feature selection is categorized into three approaches: filter, wrapper, and embedded methods [202]. The filter method selects features based on their intrinsic properties, independent of any ML model. It ranks features using a relevance score and applies a predefined threshold to determine the optimal subset. The workflow of this method is more like a ranking process, selecting the high‐scoring feature and discarding low‐scoring features [203]. The wrapper method, on the other hand, assesses subsets of features based on their effectiveness in improving a particular model. It wraps the feature selection around the learning algorithm and, using cross‐validation, then evaluates the benefits by adding or removing a feature. A commonly used wrapper method is sequential forward selection (SFS). SFS starts with an empty feature set and iteratively adds one feature at a time, selecting the feature that maximizes model accuracy at each step [204, 205]. Embedded methods integrate feature selection as part of the model training process, offering a balance between filter and wrapper methods [206, 207]. During normalization and standardization, features and target matrices are rescaled to ensure equal footing, preventing large‐magnitude features from dominating and reducing accuracy [208]. Table S3 shows some important features of perovskites.

## 6 Model Selection

Model selection is a crucial step in any ML project; it not only determines the prediction performance but also its applicability to new data. The complete procedure starts with choosing the most appropriate algorithm from a pool of candidates in different ML methods to effectively capture the fundamental patterns or features of datasets. A well‐chosen model can effectively balance bias and variance, minimizing overfitting and underfitting, thus ensuring reliable performance on unseen data. Furthermore, model selection has a significant impact on computational efficiency which is crucial for processing large datasets. Liu et al. [209] also highlighted that the model selection is one of the five main parts of a complete ML‐driven perovskite research, along with data curation, feature engineering, model validation, and interpretability. Their framework provides a valuable reference for the algorithm selection discussed in this section. In the following chapter, we mainly discuss the established ML algorithms covering supervised, unsupervised, and reinforcement learning which have been applied to perovskite materials and devices. And we also highlight the emerging trend toward advanced generative models, which represent a promising direction for the future of new materials discovery. A brief overview of more detailed ML categories and algorithms can be found in Figure S1.

### 6.1 Supervised Learning

Supervised learning is a task‐driven approach aimed at constructing a relationship between a collection of input variables (X) and an output variable (Y). This relationship is then used to predict outcomes for unknown data [210]. There is another important concept in supervised learning: Interpolation and extrapolation (as can be seen in Figure S2). Interpolation involves making predictions within the scale of the training data, the model estimates outputs for inputs that are similar or within the bounds of the data it has already seen [211]. In material science, interpolation generally corresponds to predictions made within the composition or structural space represented in the training dataset. When a model encounters compounds with similar element combinations, lattice types, or bonding environments to those it has already learned, the predictions are usually more stable and accurate. By contrast, extrapolation involves making predictions outside the domain of the training data [211]. In this case, the model's predictive reliability deteriorates due to the lack of reference data, leading to higher uncertainty and potential overfitting to complex correlations.

To tackle this issue, uncertainty quantification (UQ) methodologies, including ensemble learning [212] and Bayesian neural networks [213] have been progressively implemented to evaluate model confidence and differentiate between epistemic (model‐related) and aleatoric (data‐related) uncertainty. Adding UQ to ML‐driven screening pipelines makes predictions easier and more accurate. This helps researchers focus on candidates who are likely to perform satisfactorily with minimal uncertainty, which makes data‐driven materials discovery stronger.

#### 6.1.1 Linear Models

The linear regression model, along with its derivation, multilinear regression, stands as the most basic and extensively utilized ML model in material science, often referred to as:

(6) $$f_{w,b}\left(x\right)=\omega x+b$$

where, *f*_*w*,*b*_(*x*) abbreviated as y is the target property, x is the feature descriptor, b is the bias and ω is the regression weight which is the value to construct the relationship between y and x. Normally, feature descriptors are multiple and multidimensional which can be presented in matrix: $X=\left[\begin{array}{ccc} x_{11} & x_{12} & x_{13}\\ x_{21} & x_{22} & x_{23}\\ x_{31} & x_{32} & x_{33} \end{array}\right]$, with the corresponding target property of $y=\left[\begin{array}{c} y_{1}\\ y_{2}\\ y_{3} \end{array}\right]$, and $\omega =\left[\begin{array}{c} \omega_{1}\\ \omega_{2}\\ \omega_{3} \end{array}\right]$, then for the given data *x*_1_, that is, the first column data of the matrix X, the prediction result µ_1_ will be given by the following equation:

(7) $$\mu_{1}={\left[\begin{array}{c} x_{11}\\ x_{21}\\ x_{31} \end{array}\right]}^{T}\;*\left[\begin{array}{c} \omega_{1}\\ \omega_{2}\\ \omega_{3} \end{array}\right]$$

Logistic regression is another type of linear regression which is called generalized linear model for classification tasks. Its primary goal is to establish a regression equation for classification by defining the decision boundary using existing data [214]. It achieves this through the sigmoid function, which models conditional probability. The sigmoid function is expressed as:

(8) $$g\;\left(z\right)=\frac{1}{1+e^{-z}}$$

Where z represents either a single value or an array of values. When applied to arrays, the sigmoid function leverages vectorization, enhancing computational efficiency. The logistic regression equation applies the sigmoid function to the linear model, as follows:

(9) $$f_{w,b}\left(x\right)=g\left(wx+b\right)$$

Linear models are easy to train and interpret and can achieve good results for properties with approximately linear relationships. Li et al. [215] used ridge regression to predict the thermodynamic stability of perovskite oxides, and the results showed that ridge regression has the smallest prediction deviation for thermal stability. The advantages of linear models are simple implementation, fast speed, and interpretable results, which are suitable for small data sets or preliminary analysis. The disadvantages are that they perform poorly when there is a nonlinear relationship between properties and descriptors, and it is difficult to capture complex interactions, and artificial features need to be constructed to compensate for the underfitting of the model [216].

#### 6.1.2 Kernel‐Based Models

SVM is an advanced model, with its fundamental concept revolving around identifying the optimal hyperplane that separates the different classes in the feature space. In two‐dimensional space, this hyperplane manifests as a line, aiming to characterize the data into distinct classes. The core of an SVM lies in solving a quadratic programming problem [217] to maximize the margin between the data points and the hyperplane. The equation is:

(10) $$f_{w,b}\;\left(x\right)=w^{T}+b=\sum_{i=1}^{m}a_{i}y^{\left(i\right)}\langle x^{\left(i\right)},\;x\rangle +b$$

The 〈*x* ^(*i*)^, *x*〉 above is the Kernel function applied on input space (*x* ^(*i*)^, *x*) which can be also written as *k*(*x* ^(*i*)^, *x*). As mentioned, SVM excels in situations requiring linear two‐dimensional classification. However, when the data is not linearly separable in the original space or input space, a transformation is necessary to map the data from the original space to a higher dimensional feature space. The goal is to make the classes linearly separable in this new, high‐dimensional feature space, allowing for the fitting of decision boundaries to separate classes and make predictions. A crucial technique in SVM for addressing non‐linear data is the kernel trick. This technique allows SVM to efficiently handle non‐linear data by enabling learning algorithms to function in high‐dimensional spaces without changing the underlying algorithm. However, kernel techniques can also lead to the curse of dimensionality in some cases, especially when the feature space dimension is too high, the data becomes sparse, increasing the difficulty of model training and the risk of overfitting. In addition, the computational complexity of the kernel matrix grows quadratically with the size of the dataset, resulting in a significant increase in computational and memory costs, especially on large datasets. Improperly selected kernel functions may also make the model too complex, further exacerbating the problem of the curse of dimensionality. Therefore, when using kernel techniques, it is necessary to carefully select the kernel function and balance the data size with the model complexity to avoid the effects of the curse of dimensionality and excessive computational complexity. There is a simplified explanation of kernel trick, supposing we have data $(x^{\left(i\right)},\;x)\in X$, and there is a mapping from original feature x to high dimensional feature vector x→∅(x), then a kernel function *k* (*x*, *z*) =  ∅(*x* ^(*i*)^)^*T*^∅(*x*) can be applied to simplify the high dimensional vector.

Kernel‐Based Models can be used to predict many perovskite properties: Feng et al. [218] used SVM to predict the bandgap of HOIPs, the SVM achieved 0.974 R^2^ which showed relatively acceptable accuracy. In addition, SVM also has advantages in predicting crystal structure and lattice parameters. Jarin et al. [219] used genetic algorithm‐supported SVM to achieve 87% accuracy in predicting crystal structure type (including Cubic, orthorhombic, tetragonal and orthorhombic crystal systems) and 95% accuracy in predicting lattice parameters. On the other hand, kernel ridge regression also achieved high accuracy with 0.854 R^2^ in the study of ABX_3_ perovskite bandgap [220]. The kernel‐based model can effectively handle nonlinear relationships, is robust in the case of high‐dimensional feature space and small samples and can avoid data overfitting. However, the model parameters (such as kernel function type, penalty coefficient, etc.) need to be carefully adjusted, the training speed is not ideal for large data sets, and the model results are not as intuitive as linear models and have weaker interpretability [221].

#### 6.1.3 Tree‐Based Models

Tree‐based models represent a significant category of ML algorithms, built upon the foundation of decision trees. These models are widely utilized for both classification and regression tasks. The core mechanism of tree‐based models involves performing conditional judgments on data features to recursively partition the dataset into smaller subsets, ultimately forming a tree‐structured decision framework [222]. The construction of decision trees is grounded in the principles of nodes and splits. Each internal node represents a test on a specific feature, while each leaf node corresponds to a class label or a numerical prediction. The dataset is split into subsets by selecting the optimal feature and its corresponding threshold. This splitting process is guided by three criteria: Information Gain, Gini Index, and Reduction in Variance [223]. The splitting is recursively applied to each subset until a stopping condition is met, such as reaching a maximum depth, a minimum sample size, etc. In addition to decision trees, most traditional models currently applied in the field of materials science are also tree‐based models. These include algorithms such as Random Forest [224], LightGBM [225], and eXtreme Gradient Boosting (XGBoost) [226], which have become widely utilized due to their robustness and effectiveness in handling complex datasets.

Tree‐based models are widely used in predicting PV material properties and often achieve high accuracy. Eren et al. [227] used the random forest model to predict the bandgap and device PCE of perovskites, and achieved high‐precision fitting of both results for a small amount of experimental data (8 compositions) (R^2^> 0.99 for absorption spectrum data and R^2^> 0.82 for *J*–*V* characteristic data), which can accurately reproduce the experimental values. On the other hand, tree‐based models are also used to predict the band structure of perovskites. Mattur et al. [173] used RF to classify perovskite oxides as direct bandgap or indirect bandgap. The RF model achieved an accuracy of about 91%. Liu et al. [228] compared LR, KNN, SVR, RF, MLP and XGBoost to predict the bandgap of ABX_3_ type perovskites based on 227 sets of experimentally measured data collected from 1254 publications. XGBoost model achieved the highest predictive accuracy (RMSE = 0.055 eV, R = 0.99) while RF model also achieved high accuracy (RMSE = 0.064 eV, R = 0.98). Even compared to currently mainstream neural networks, tree models still perform well. Zhu et al. [229] compared ANN, GBDT, KNN, RF, and XGBoost to predict the decomposition energy, bandgap, and spectrally limited maximum efficiency (SLME) of 177 264 halide perovskites. The XGBoost model achieved the best performance, with an RMSE of 0.19 eV for decomposition energy, 0.20 eV for bandgap, and 1.77% for SLME, demonstrating high prediction accuracy and generalization capability. The advantages of tree‐based models include being able to automatically characterize high‐dimensional nonlinear relationships, being generally insensitive to input feature distributions, and having good robustness to small and medium‐sized data. In addition, random forests and other models have low risks of noise and overfitting and can provide a robust benchmark model. However, decision tree models are more dependent on data quality, and data bias may affect model generalization. Boosting models have more hyperparameters and require sufficient tuning to prevent overfitting. Compared with simple models, the results of tree models are difficult to directly parse into physical meaning (explanation methods are required). Nevertheless, the balance between comprehensive accuracy and interpretability makes tree models one of the common choices for predicting the properties of perovskite materials [230, 231].

#### 6.1.4 Deep Learning

**FIGURE 8:**
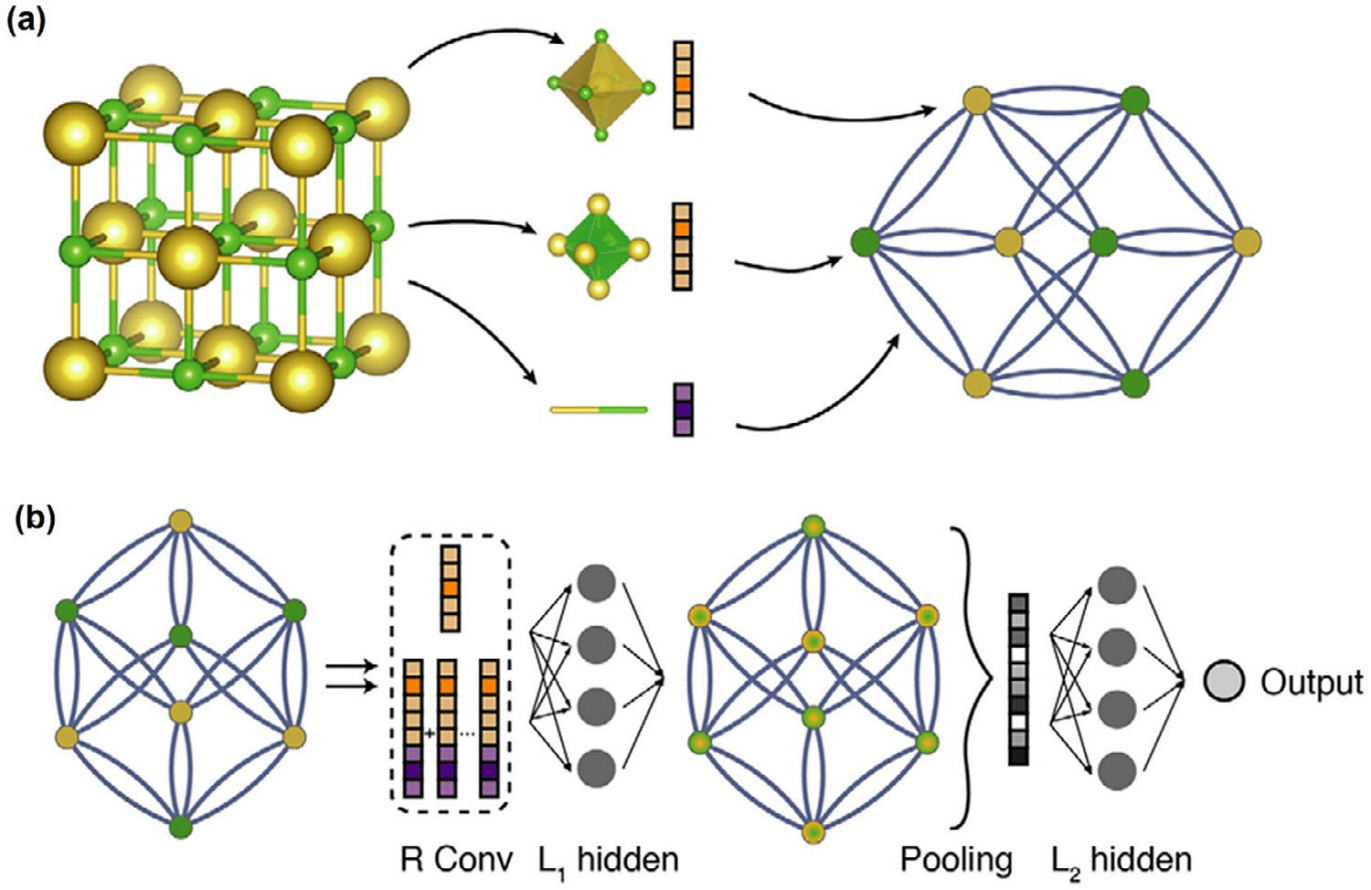
Illustration of the crystal graph convolutional neural networks. (a) Construction of the crystal graph. (b) Structure of the convolutional neural network on top of the crystal graph. Reproduced with permission [232]. Copyright 2018, Physical Review Letters.

Deep learning extracts feature relationships through multi‐layer neural networks and have strong nonlinear fitting capabilities. In the research of perovskite materials, deep models have begun to emerge in recent years [22]. Multilayer feedforward neural networks (also known as ANNs or BPNNs) have demonstrated significant effectiveness in predicting various properties of perovskites. For example, a previous study successfully developed a BPNN model to simultaneously predict multiple key properties of perovskite oxides, including formation energy, thermodynamic stability, unit cell volume, and oxygen vacancy formation energy. The results indicated that the neural network achieved low prediction errors across all these properties, particularly excelling in the prediction of oxygen vacancy formation energy, outperforming other models tested. In contrast, other simple models have their own strengths (e.g. RF is better at forming energy, SVM is better at volume), while deep neural networks have achieved higher accuracy in all indicators [215]. This shows that deep learning models can capture complex mappings of different properties at the same time. In addition, deep learning can also be combined with more abstract descriptions, such as directly using atomic composition and structure as input. A representative example is the graph neural network (GNN) approach, particularly the Crystal Graph Convolutional Neural Network (CGCNN), which has shown strong performance in materials research [232]. CGCNN treats a crystal as a graph in which atoms act as nodes and bonds as edges, enabling the model to learn structure–property relationships directly without using predefined descriptors. Through convolutional operations on atomic graphs, it captures both local coordination and long‐range interactions. This method would be especially useful for complex systems like PIMs, where atomic arrangement and mixed‐anion coordination strongly influence stability and optoelectronic behavior [20]. Figure 8 illustrates the working mechanism of CGCNN.

In the field of perovskite solar cells, many major research efforts have employed neural network to simulate complicated device behaviors and performance relationships. Liu et al. [233] combined convolutional neural network (CNN) and RF with SHapley Additive exPlanations (SHAP) and Genetic Programming Symbolic Regression (GPSR) to predict both photovoltaic parameters (PCE, *J*_sc_, *V*_oc_, FF) and EQE spectra with high accuracy (RMSE ≤ 1.25%). Yan et al. [234] established an interpretable RF framework combined with SHAP to find a link between experimental characterization data and photovoltaic performance. The model accurately reproduced both the bandgap (R^2^ > 0.99) and PCE (R^2^ > 0.82) using optical absorption and *J*–*V* curves from eight perovskite compositions as input. The SHAP‐based feature attribution further identified that the absorption edge (∼750 nm) and *J*–*V* slope are the most influential factors governing PCE, demonstrating that ML can serve as a “virtual characterization tool” directly linking spectral features to device efficiency with excellent precision. Liu et al. [235] employed Random Forest (RF) and XGBoost models integrated with SHAP to predict multiple key photovoltaic parameters of perovskite solar cells, including PCE, *V*_oc_, *J*_sc_, and FF, based on 814 experimentally validated datasets. The optimized RF model achieved outstanding outcomes, with RMSE values of 1.58% (PCE), 0.051 V (*V*_oc_), 1.04 mA cm^−^^2^ (*J*_sc_), and 0.046 (FF), and correlation coefficients (r) above 0.85 for most targets. Moreover, SHAP analysis indicated that a moderate bandgap (1.25–1.5 eV), an appropriate energy offset (∼0.15–0.2 eV), and elevated carrier mobility are essential factors that improve device efficiency, providing robust physical interpretability and alignment with the SQ limit.

**FIGURE 9:**
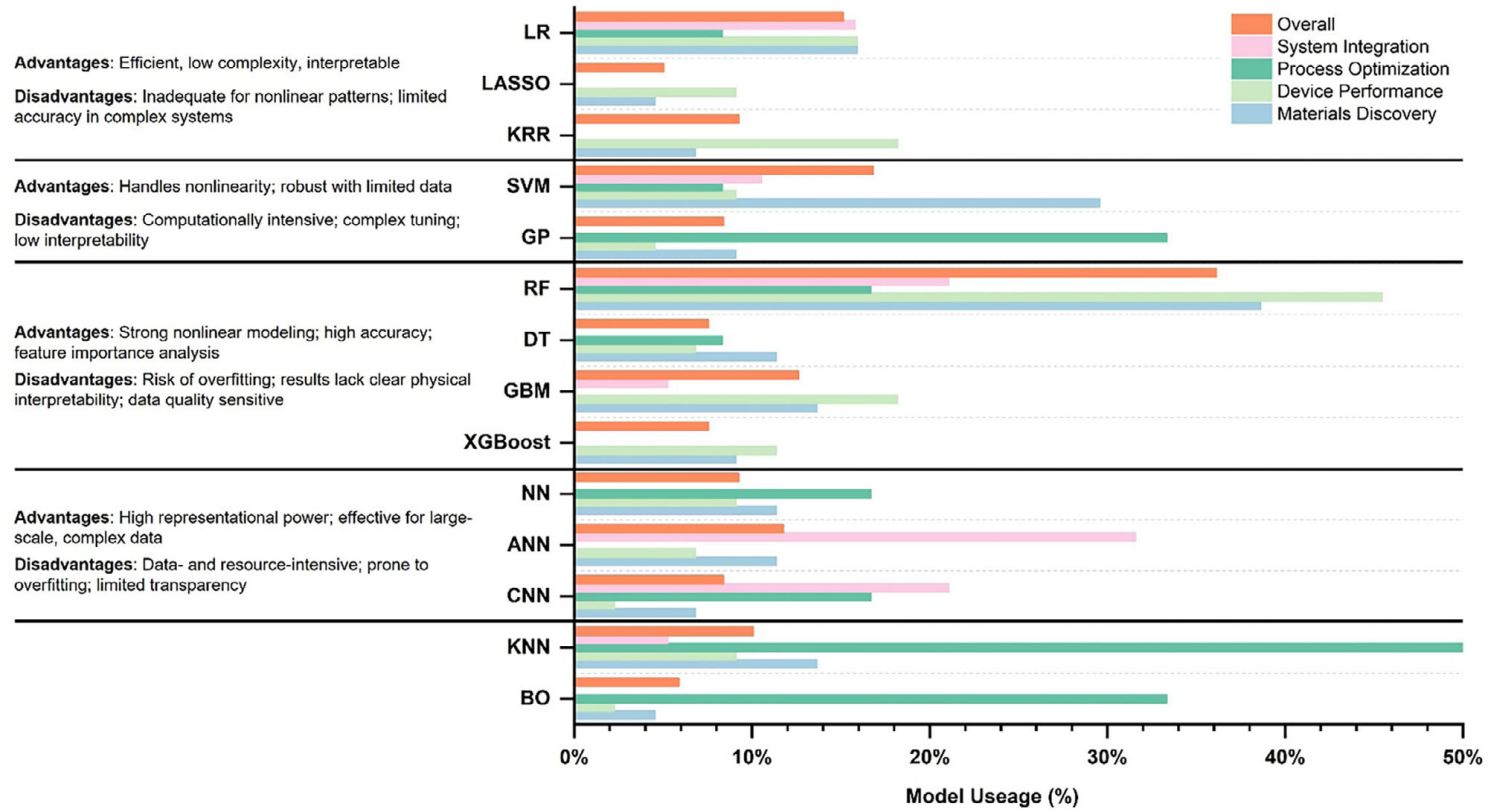
The usage of different algorithms in 119 perovskite solar cell research papers reproduced with permission [236]. Copyright 2025, Elsevier.

The advantages of deep learning are that it can automatically extract complex patterns from big data without the need to manually set specific nonlinear forms. Especially when the amount of data is large enough (for example, a perovskite database containing tens of thousands of samples), deep models are expected to further improve prediction accuracy. At the same time, deep models are easy to combine with physical priors (such as combining SQ limit to verify the relationship between bandgap and device parameters and used for joint optimization of materials and devices). Its disadvantage is that it has high requirements for data volume and quality. If the deep model is not trained with enough samples, it is easy to overfit, resulting in inaccurate predictions of unknown combinations. In addition, deep learning are also “black box” models that lack intuitive interpretability and require additional methods (such as feature visualization and sensitivity analysis) to explain their decision‐making basis. Therefore, for materials with limited data, such as PIMs, it is often necessary to carefully evaluate whether to use complex neural networks or data augmentation to ensure model reliability. Figure 9 (collected from ref [236]) summarises the usage of different ML algorithms on perovskite solar cell research and highlights the advantages and disadvantages of four main classes of supervised learning.

### 6.2 Unsupervised Learning

Unsupervised learning, another important type of ML, involves analysing data without predetermined labels or categories. This approach enables computers to independently discover inherent patterns and categorizations within the data [237]. This method is based on the principle of identifying inherent structures in data, which is a key concept in the field of AI. It deviates from supervised learning, which depends on labelled datasets to train models [238]. The theoretical foundation of unsupervised learning revolves around the assumption that datasets, regardless of human‐imposed classifications, inherently exhibit inherent clusters or patterns that can be identified by algorithmic analysis [239]. Principal component analysis (PCA) and word embedding methods are two main approaches used for discovering solar materials and PIMs. PCA is a statistical dimensionality reduction technique that transforms complex, high‐dimensional datasets into a smaller set of uncorrelated variables, known as principal components, effectively capturing most of the variance in the original dataset. However, this method naturally has the risk of information loss, so it is necessary to carefully select the appropriate dimension during the dimensionality reduction process to achieve the best balance between information retention and model complexity [240, 241]. Hosni et al. [242] employed PCA to systematically reduce the dimensionality of perovskite materials data to optimize ML predictions of specific surface area, a critical factor influencing PV performance. The results show that PCA can significantly improve the prediction performance of the model in both the RF model and the SVR model. The difference is that in the SVR model, when the feature dimension reaches five, the model performance tends to be stable, but in the RF model, when the dimension reaches the optimal state (nine dimension), there is a downward trend. This result shows that PCA may be more suitable for algorithms that is sensitive to linear relationships in data. In addition, Mai et al. [243] employed PCA for dimensionality reduction in their ML work aimed at identifying organic spacer materials (ammonium salts) that could significantly enhance the PCE of perovskite solar cells. By reducing the original 16‐dimensional feature space to just two principal components, PCA substantially simplified the complexity of the predictive model. This dimensionality reduction not only streamlined the data representation but also markedly improved the model's predictive accuracy and generalization to novel, unexplored materials. In addition to PCA, word embedding techniques, rooted in natural language processing (NLP), have been effectively applied to mining and predicting PV materials from extensive literature databases. Zhang and He [244] utilized an NLP‐based unsupervised learning method, leveraging word embedding models to automatically extract hidden relationships between material compositions and their PV properties from textual databases, and successfully output well‐known solar materials including Si, GaAs, ZnO, CIGS, InP, c‐Si, CdS, GaInP, and InGaAsP. In addition to common solar cell materials widely reported in the literature, the NLP‐based approach also predicted several uncommon materials, such as As_2_O_5_; the optoelectronic properties of As_2_O_5_ were further investigated through first‐principles calculations, and the accuracy of the ML predictions was confirmed. Expanding the scope of word embedding techniques, Zhang et al. [245] developed a new NLP model specifically for non‐English scientific literature. They constructed a substantial database comprising 210 000 Chinese‐language abstracts on materials and chemical research. The model successfully screens out materials that are highly relevant to “solar cells” from the literature, identifies known materials (such as CH_3_NH_3_PbX_3_), and predicts potential new candidates (such as TiNb_2_O_7_, BiPO_4_, Y_2_O_3_). These new candidates were further verified by DFT under three evaluation metrics include bandgap (1.6 eV for TiNb_2_O_7_ and Y_2_O_3_, 2.1 eV for BiPO_4_), UV–vis absorption spectrum and three theoretical efficiencies: spectrally limited maximum efficiency (SLME, 26.7–28.1%), SQ limit (20.7‐3‐0.6%) and “potential energy loss” maximum achievable efficiency (16.0–24.9%). The results confirm the potential of these materials for solar cell applications.

### 6.3 Reinforcement Learning

Reinforcement Learning (RL) is a special type of ML where an agent learns to make decisions by carrying out actions in an environment towards achieving some goals. What sets RL algorithms apart is that they can learn what the best actions are through the experience of interaction with the environment, not learning from a pointed set of datasets [246, 247]. One of the main parts of RL is the policy, which allows the agent to execute the next step based on its current stage. Algorithms used in RL mainly fall into three classes: (1) policy‐based, (2) value‐based, and (3) model‐based methods. Regarding policy‐based methods, it directly learns the policy function that maps to state the probability of taking each action. The advantage of policy‐based methods is their effectiveness in high‐dimensional or continuous action spaces [248]. Value‐based methods, like Q‐learning and Deep Q‐Networks, learn the value of each action in a state from experiences and choose actions based on the values learned [249]. In contrast to policy‐based methods, value‐based methods focus on learning the value of each action in a given state rather than directly learning the policy. They are simpler and can be more effective when dealing with discrete action space [250]. Model‐based methods attempt to model the environment and then plan the workings of the model; they can be more sample efficient than model‐free methods (like policy‐ and value‐based), as they can use their model to simulate experiences rather than having to actually experience them [251, 252]. Currently, there is limited report on the application of RL in the field of material exploration. It is usually used for the optimization design of PV devices. For example, Jiang and Yoshie [253] proposed an RL method. The high‐dimensional refractive index data of more than 300 materials were reduced to a two‐dimensional environment space, and the materials and thickness of the multilayer solar absorber were automatically optimized. After 1000 iterations, the method designed a 5‐layer structure composed of MgF_2_, TiO_2_, Si, Ge, and Cu, with thicknesses ranging from 35.3 to 200.0 nm, and achieved an average absorption rate of 91% in the 250–800 nm band. On the other hand, Sajedian et al. [254] used a dual deep Q network to optimize a symmetric metamaterial consisting of a cylinder and two layers of film, generating 1250 perfect solar absorbers in about 35 000 steps from about 527 billion designs. The absorption rate of these structures in the 350–800 nm band exceeds 90% (up to 97.6%) and found the absorption rate in the 8–13 µm band is less than 10% (minimum 1.37%). These two studies demonstrate the application of RL in the efficient exploration of the design space of solar energy and perovskite‐related materials, respectively improving the absorption performance through multi‐material selection and structural optimisation and providing a new path for automated design in the PV field.

### 6.4 Generative Models

Generative models have recently become a breakthrough method in material science [255, 256]. They make it possible to inverse design new materials including perovskite and PIMs. Generative models are different from traditional supervised models, they attempt to understand the fundamental probability distribution of materials data, thereby enabling the independent creation of chemically valid structures with specified physicochemical properties. This ability makes it possible to systematically explore areas of compositional and structural space that have never been explored before, which can definitely accelerate the discovery of new materials. Currently, there are two main branches of generative models: Large Language Models (LLMs) and Diffusion Models.

**FIGURE 10:**
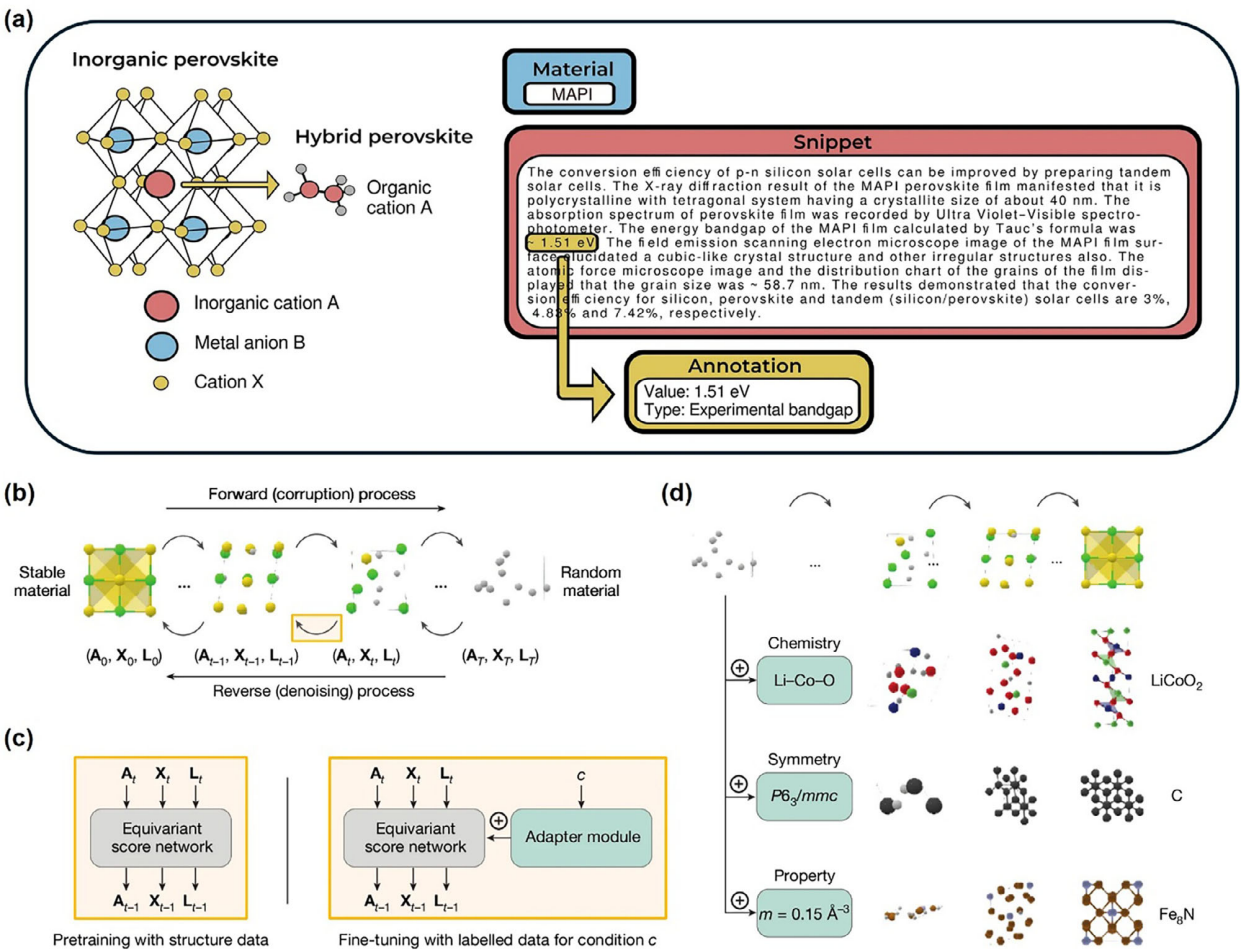
Illustration of generative models. (a) Demonstration of key information extraction by LLMs reproduced under terms of the CC‐BY license [257]. Copyright 2025, The Authors, Springer Nature. (b–d) Inorganic materials design with MatterGen reproduced under terms of the CC‐BY license [258]. Copyright 2025, The Authors, Springer Nature.

LLMs rely on autoregressive token prediction, where the model learns contextual dependencies within sequential data to predict the next word or token, thereby capturing high‐dimensional relationships between syntax, semantics, and scientific knowledge. When trained on scientific papers, this mechanism enables the LLM to learn the co‐occurrence patterns between materials, properties, and numerical values that appear in research papers. As a result, LLMs can effectively capture structure–composition‐property relationships and extract key information such as bandgap and *E_hull_* directly from literature. In this way, LLMs can rapidly construct reliable, experiment‐based databases by capturing and extracting key information from vast scientific literature, effectively addressing the long‐standing issue of data scarcity. Sipilä et al. [257] demonstrated the practical application of LLM in helping constructing PV600 database which contains bandgaps information of HOIPs, by extracting information from 238 431 open access paper. Figure 10a shows the conception of information extraction from literature.

Diffusion models, on the other hand, learn how to turn Gaussian noise into realistic crystal structures by denoising them over and over. This gives them strong generative stability and structural diversity. Recent frameworks like MatterGen [258] has shown that it can make inorganic materials with lattice symmetry, formation energy, and electronic structure that can be controlled. MatterGen combines a crystal graph encoder with a denoising diffusion probabilistic model to sample atomic positions and lattice parameters at the same time. This makes it the best tool for structural validity and property control. Figure 10b–d demonstrates the complete inorganic material design workflow of MatterGen.

Although the application of generative models on perovskites and PIMs is still developing, these methods have already shown great potential for solving one of the biggest problems in material science: the lack of high‐quality large datasets. We believe that generative models will become a more reliable model development direction for material science in the future.

## 7 Model Optimization and Evaluation

During model training, the optimal values of parameters ω and b are typically determined by minimizing the discrepancy between the predicted output µ and the actual output y. This discrepancy is quantified through a loss function. For linear regression, the Mean Squared Error (MSE) is commonly employed:

(11) $$J_{\left(w,b\right)}=\frac{1}{2m}\sum_{i=0}^{m-1}{\left(f_{\left(\omega ,b\right)}\left(x^{\left(i\right)}\right)-y^{\left(i\right)}\right)}^{2}$$

Where m is the number of training examples. One problem with linear regression is the possibility of underfitting because it seeks the unbiased estimate with the smallest MSE. If the model is underfitted, it will not be able to achieve good prediction results [259]. This occurs because linear regression seeks an unbiased estimate with the lowest MSE, which might not be flexible enough for complex data. To address this, certain regularization techniques, such as Ridge or Lasso regression, intentionally introduce a small amount of bias to the model, which can help reduce variance and improve predictive accuracy.

Gradient descent is an optimization method widely used to minimize the loss function by iteratively updating parameters [260]. The equation of gradient descent is listed below, and where λ is the learning rate:

(12) $$\begin{array}{cc} b & =b-\;\lambda \frac{\partial J_{\left(w,b\right)}}{\partial_{b}}\\ \omega & =\omega -\;\lambda \frac{\partial J_{\left(w,b\right)}}{\partial_{\omega }} \end{array}$$

Coefficient ω and b will keep updating simultaneously, where:

(13) $$\frac{\partial J_{\left(w,b\right)}}{\partial_{b}}=\frac{1}{m}\sum_{i=0}^{m-1}\left(f_{\left(\omega ,b\right)}\left(x^{\left(i\right)}\right)-y^{\left(i\right)}\right)$$

(14) $$\frac{\partial J_{\left(w,b\right)}}{\partial_{\omega }}=\frac{1}{m}\sum_{i=0}^{m-1}\left(f_{\left(\omega ,b\right)}\left(x^{\left(i\right)}\right)-y^{\left(i\right)}\right)x^{\left(i\right)}$$

In the gradient descent, the choice of λ is crucial, when the value of λ is too big, the coefficient may not converge. If the λ is too small, the gradient descent can be very time‐consuming or even get stuck in a plateau. Thus, careful tuning of the learning rate is necessary for efficient convergence.

For logistic regression, the commonly used loss function is cross‐entropy loss, suitable for classification tasks, where predictions are probabilities between 0 and 1:

(15) $$J\left(w,b\right)=\frac{1}{m}\sum_{i=0}^{m-1}\left[Loss\left(f_{w,b}\left(x^{\left(i\right)}\right),y^{\left(i\right)}\right)\right]$$

(16) $$Loss=\left(-y^{\left(i\right)}\log\left(f_{w,b}(x^{\left(i\right)}\right)\right)\;-\left(1-y^{\left(i\right)}\right)\log\left(1-f_{w,b}\left(x^{\left(i\right)}\right)\right)$$

Moreover, cross‐validation and grid search are two commonly used hyperparameter tuning (optimizing) methods while performance metrics is for evaluation. The hyperparameter in ML refers to a parameter whose value is set before the learning process begins, as opposed to parameters that are learned from the data during training. Hyperparameters control the overall behavior of the learning algorithm, influencing how the model learns and performs [261]. Cross‐validation (CV) is a commonly used technique for evaluating the prediction accuracy of statistical models, especially when it comes to selecting the best model and fine‐tuning its hyperparameters [262]. The fundamental logic of CV is partitioning the data into some subsets, training the model using certain subsets, and then verifying it using the remaining part. There are many CV schemes, such as k‐fold CV, leave‐one‐out (LOO), and blocked CV. K‐fold is one of the most used methods, this is because the data sets in materials science are usually limited in size, and K‐fold cross‐validation can provide more stable and reliable performance evaluation on limited data. At the same time, the computational cost of K‐fold cross‐validation is relatively low and can be effectively implemented in practical applications. In addition, k‐fold cross‐validation can more comprehensively evaluate the stability and generalization performance of the model by splitting and validating the data multiple times [263]. LOO is an extreme case of K‐Fold, where K is equal to the size of the dataset. In each iteration, only one data point is used as the validation set, and the remaining data points are used as the training set. LOO can maximize the use of data, but the computational cost is high, especially for large datasets [264]. Blocked CV is always applied in dealing with time series data or data with a natural order, mainly used to process batch experimental data: for example, material samples produced in different batches may have different properties due to batch differences [265]. Therefore, it is not as versatile as k‐fold in the field of material science. Grid Search is another important hyperparameter optimization technique widely used in ML. It systematically searches every combination in a predefined parameter space to find the parameter settings that optimize the model performance. Different models have different hyperparameters, and these hyperparameters have a significant impact on the performance of the model [266]. For example, in SVM model, it can optimize the regularization parameter C and kernel function parameters [267], in the random forest algorithm, it can optimize the number of trees (n_estimators), maximum depth (max_depth), the minimum number of sample leaf nodes (min_samples_leaf), etc [268]. And optimizing learning rate, number and size of hidden layers, activation functions, etc. in neural networks [269]. Performance metrics are used to evaluate and compare model performance. Commonly used metrics include MSE, percentage of absolute difference (PAD%), accuracy, Precision, Recall, F1‐score, coefficient of determination (R^2^), Pearson coefficient (r), and Area Under the Receiver Operating Characteristic curve (AUC‐ROC). The explanations of these metrics can be found in Equations (11), (17)–(19) for regression and (15), (20)–(23) for classification.

(17) $$PAD\;\left(\%\right)=\frac{\left|experimental-predicted\right|}{experimental}*100$$

(18) $$R^{2}=1-\frac{\sum_{i}{\left(y_{i}^{ture}-y_{i}^{pred}\right)}^{2}}{\sum_{i}{\left(y_{i}^{ture}-{\bar{y}}_{i}^{true}\right)}^{2}}$$

(19) $$r^{2}=\frac{\sum_{i}\left(y_{i}^{ture}-{\bar{y}}_{i}^{true}\right)\left(y_{i}^{pred}-{\bar{y}}_{i}^{pred}\right)}{\sqrt{{\sum_{i}{\left(y_{i}^{ture}-{\bar{y}}_{i}^{true}\right)}^{2}\left(y_{i}^{pred}-{\bar{y}}_{i}^{pred}\right)}^{2}}}$$

(20) $$Accuracy=\frac{TP+TN}{TP+FP+TN+FN}$$

(21) $$Precision=\frac{TP}{TP+FP}$$

(22) $$Recall=\frac{TP}{TP+FN}$$

(23) $$F_{1}=\frac{2\times Precision\times Recall}{Precision+Recall}$$

Where TP is the true positive rate, FP is the false positive rate, TN is the true negative rate, and FN is the false negative rate.

## 8 Model Interpretability

With the development of ML models, the complexity of models is also increasing. Being able to understand them has become an important part in material science. This makes sure that predictions are not only correct but also has certain physical meaning. Interpretability connects data‐driven correlations with a basic understanding of physics. This makes it possible to find structure‐property relationships that can help with smart material design. In this section, we summarize important interpretability frameworks, such as SHAP, symbolic regression, and attention mechanisms. These frameworks go beyond just making predictions to give us mechanistic and quantifiable information about how materials behave.

### 8.1 SHAP

**FIGURE 11:**
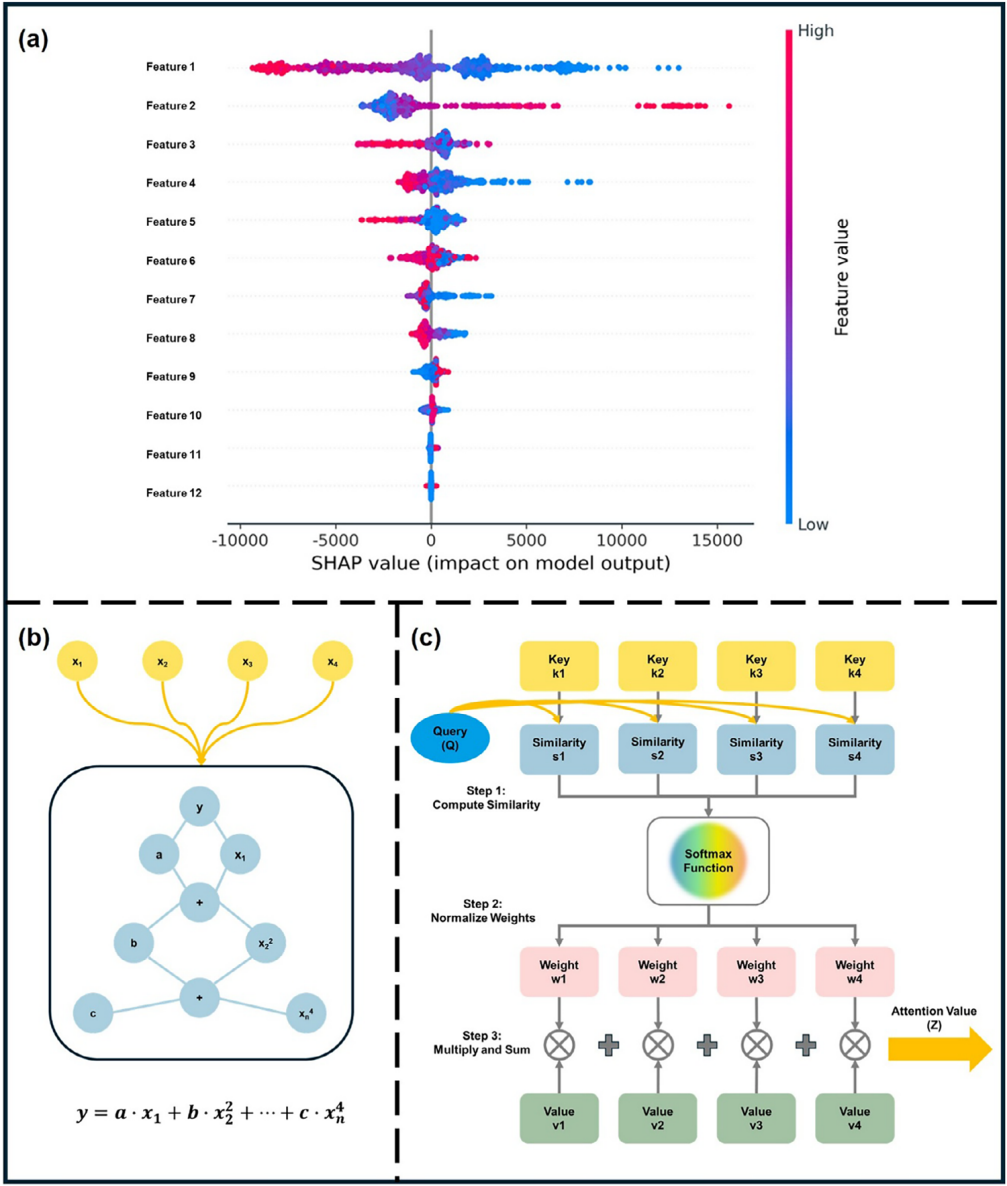
Illustration of three representative interpretability techniques. (a) SHAP Beeswarm plot [270]. (b) Symbolic regression. (c) Attention mechanisms.

Among interpretability techniques, SHAP has become one of the most powerful and widely used approaches for quantifying feature contributions in complex models. SHAP attributes a model's output to individual features by estimating their marginal contribution relative to all possible feature combinations. This additive explanation ensures both local consistency (for a single prediction) and global interpretability (across the dataset) [270]. In materials science, SHAP allows researchers to rank and see how various structural, compositional, and electronic descriptors affect a property of interest. For example, SHAP has shown that ionic radii, electronegativity differences, and tolerance factors have the most effect on the bandgap, formation energy, and thermodynamic stability of perovskite [123, 271]. Apart from feature ranking, SHAP is also able to capture the nonlinear relationship among features by dependence plots and interaction values which greatly reduce the loss of information caused by directly removing correlated features. Figure 11a shows SHAP quantitatively attributes the model output to each feature by evaluating its marginal contribution across all possible feature combinations. The horizontal distribution of SHAP values indicates both the direction and magnitude of each feature's influence, where red and blue represent high and low feature values, respectively.

### 8.2 Symbolic Regression

Symbolic regression, on the other hand, tries to find models that are straightforward to understand. Instead of trying to provide meaning of a trained model, symbolic regression looks for a clear, short, and human‐readable mathematical expression that fits the data during training. This mathematical equation (for example $y=a\cdot x_{1}+b\cdot x_{2}^{2}+\cdots +c\cdot x_{n}^{4}$) is itself a model, thus providing global interpretability. Symbolic regression creates a white‐box model that lets users see how the input features affect the output by showing them how they are related mathematically. This is particularly useful in material science and other scientific research areas that need to find underlying mechanisms or physical laws. It is a method that truly ensures interpretability from the source of model construction. Figure 11b describes the process of symbolic regression, where the algorithm constructs an analytical equation tree that directly represents the mathematical relationship between features and output, thereby offering a transparent and physically meaningful model.

### 8.3 Attention Mechanisms

Unlike SHAP, which explains predictions after a model is trained, or symbolic regression, which explicitly discovers mathematical relationships, attention mechanisms provide intrinsic interpretability within deep learning models [272, 273]. Attention does not analyse the model after training; instead, it dynamically learns the importance of each input for making a prediction during training. It does this by calculating a similarity score for each input or connection and converting it into attention weights using the Softmax function [274]. The Softmax function normalizes these scores so that their sum is 1, visually demonstrating the relative importance of each input feature or atomic environment relative to the others. The attention mechanism enables ANNs to focus on the most critical parts of the data while deemphasizing other information. Figure 11c shows the workflow of an attention mechanism, in which the model computes similarity scores between query and key vectors, applies a Softmax normalization to obtain attention weights, and aggregates the weighted values to form the attention output.

## 9 Applications of ML in Perovskite and Perovskite Inspired Materials

ML has significantly impacted materials science, especially in new materials discovery. One of the earliest notable applications of ML in this field was during the 1960s with the DENDRAL project, which was among the first computer programs designed for chemical synthesis prediction [275]. In recent years, ML has also significantly contributed to exploring and optimizing energy materials, particularly in developing high‐efficiency halide perovskite solar cells [276]. The achievements in this area primarily focus on using ML algorithms to predict material properties, including bandgap, formation energy, thermodynamic stability, crystal volume, defect energy level, etc., that are crucial for enhancing solar cell performance [215, 277]. This section highlights successful ML applications in exploring various perovskite materials and PIMs with their properties.

### 9.1 Inorganic Perovskites

**FIGURE 12:**
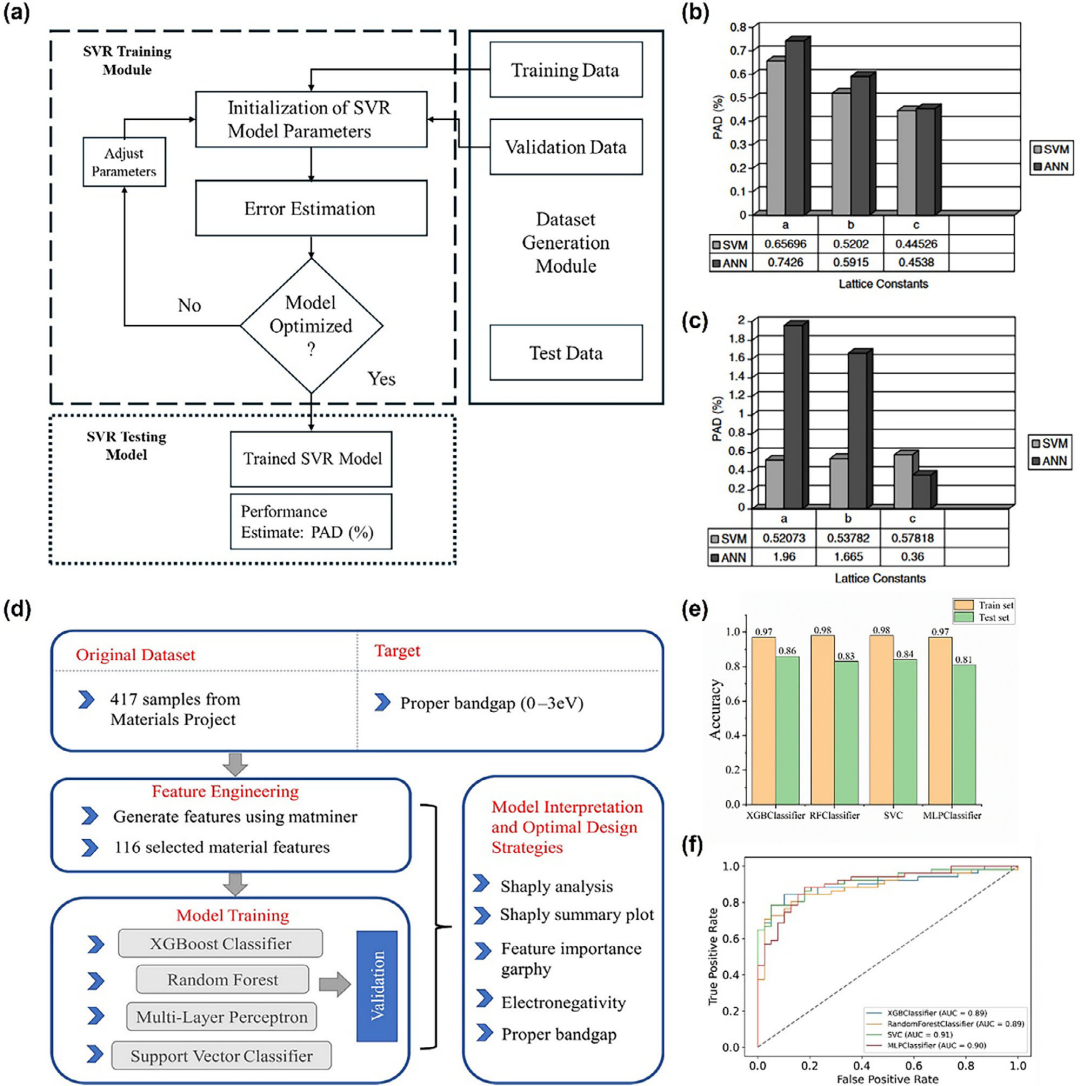
Lattice constant prediction (a–c) reproduced with permission [278]. Copyright 2007, Elsevier, and narrow‐bandgap prediction of inorganic perovskites (d–f) reproduced with permission [283]. Copyright 2024, Springer Nature. (a) The complete workflow of the SVR model. (b) Average PAD performance of ANN and SVR models on training set. (c) Average PAD performance of ANN and SVR model on testing set. (d) The ML workflow for narrow‐bandgap prediction. (e) Accuracy comparison of four algorithms on training and testing set. (f) AUC‐ROC for four algorithms on the testing set.

Earlier research on perovskite using ML dates back to 2007; Javed et al. [278] reported their work using ML on lattice constant prediction of orthorhombic ABO_3_ perovskites. SVR algorithm was used in their work; the final model has the versatility of the prediction of the lattice constant of other structurally known perovskites. As a regression work, there were five features used as input patterns, which are ionic radii of cation A, r_A_ and B, r_B_, electronegativity of cation A, x_A_ and B, x_B_, and valence of cation A, z_A_, while the target was experimental lattice constants a,b or c. In the model training stage, datasets were separated into a training set and testing set first; then, the training set was further divided into a validation set and training set; this step was achieved by cross‐validation under four‐fold [279]. ANN was also used with its predicted time cost and accuracy compared against the SVR (ground‐truth) values. In terms of time cost, the smallest gap was observed in the test group for the b lattice constant (the SVR's performance was twice that of the ANN), whereas the largest gap appeared in the c lattice constant training group (nearly a 100‐fold difference). For prediction performance, the coefficient of result accuracy (PAD) was used. Across all three lattice constants, the average PAD remained under 0.7%, significantly outperforming the ANN's predictions. The complete workflow of this work can be seen from Figure 12a, while the model performance comparison can be seen from Figure 12b,c. Although this work was done very long ago, the ML algorithm, data splitting method, and overall process are still widely used nowadays.

In recent years, numerous studies have combined ML with research on inorganic perovskites, employing various algorithms to focus on different properties such as ionic conductivity [280], dielectric breakdown strength [281], and maximum magnetic entropy change [282]. But most ML work on perovskites still centers on bandgap prediction. In 2024, Li et al. [283] proposed a method by using the XGBoost algorithm to predict the narrow bandgap range of inorganic halide perovskites. Data for 447 ABX_3_‐type inorganic halide perovskites were collected from the Materials Project database, covering the bandgap range of 0 to 6 eV, and coded samples with bandgaps between 0 and 3 eV as Samples from 1, 3 to 6 eV are coded as 0, and the data set is divided into a training set and a test set at a ratio of 4:1. In feature engineering stage, by using the Matminer tool, more additional features were generated. Afterward, the researchers performed data standardization to eliminate unit differences between features and identified and removed highly correlated features by calculating the Pearson correlation coefficient [284], ultimately retaining 116 valid features. for model training. The research team selected four classification algorithms: XGBoost, Support Vector Machine (SVC), Multilayer Perceptron (MLP), and RF, and optimized the hyperparameters of these algorithms through ten‐fold cross‐validation and RandomizedSearchCV to improve predictions accuracy and generalization ability of the model. Model performance was evaluated through indicators such as AUC‐ROC, accuracy, precision, recall, and F1 score. The results showed that the XGBoost model performed best on the test set, with an accuracy of 95%. To further interpret the model output, the researchers used SHAP [285] and identified the electron negativity range as an important factor affecting the bandgap. The larger the electron negativity range, the higher the possibility that the perovskite has a narrow bandgap. Compared to this work and the past ML work, we can find that there are many stages have been optimized, such as the application of Matminer for feature engineering, cross‐validation with more folds, hyperparameter optimization using RandomizedSearchCV (random sampling of parameter combinations which is more efficient than Grid Search where the parameter space is large and the optimal parameter range is uncertain.) and a more comprehensive evaluation of the ML model. The complete workflow can be seen in Figure 12d, and the performance of XGBoost and comparison with other models can be seen in Figure 12e,f.

### 9.2 HOIPs

**FIGURE 13:**
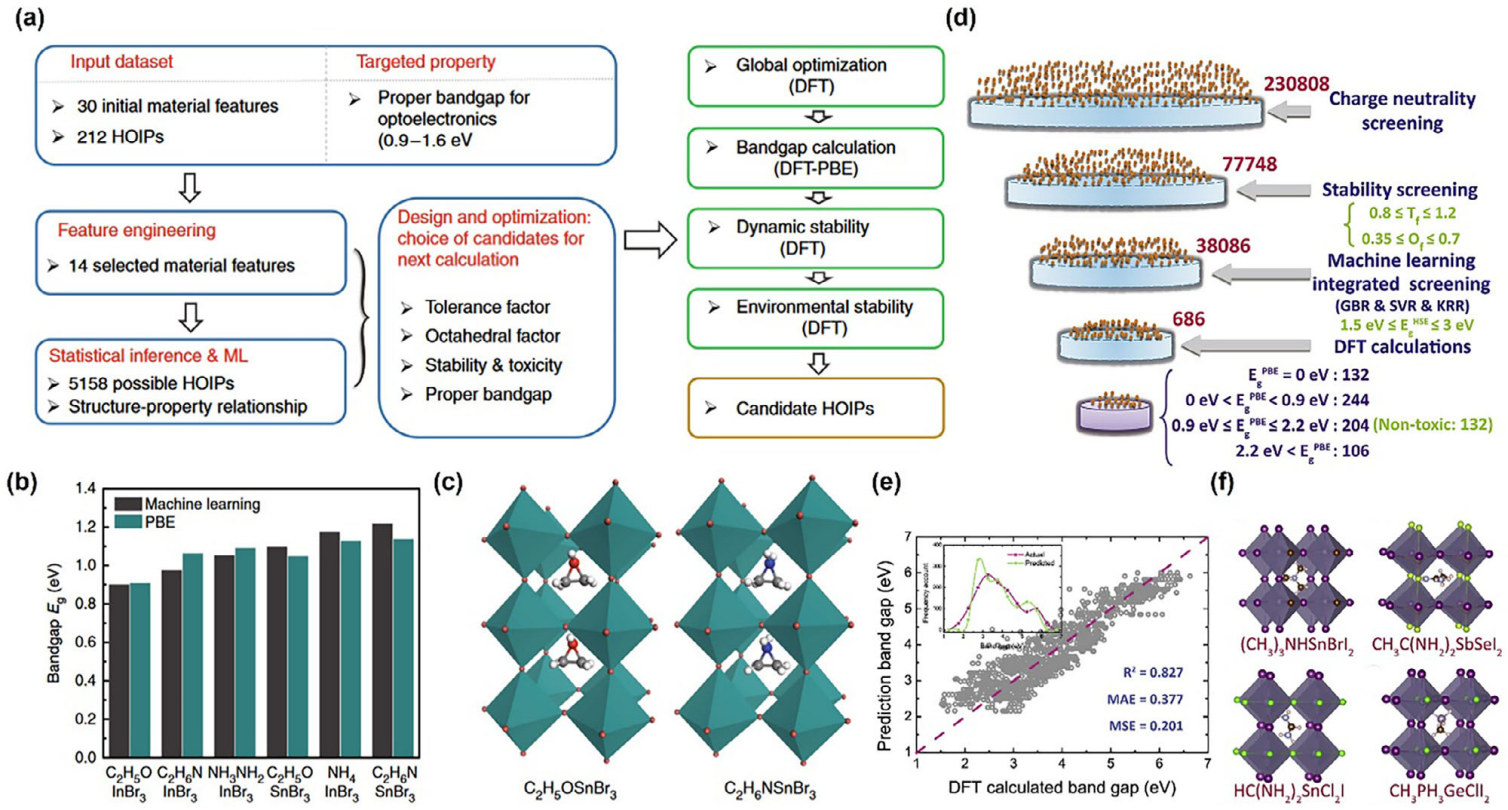
Bandgap prediction of HOIPs (a–c) reproduced under terms of the CC‐BY license [286]. Copyright 2018, The Authors, published by Nature Publishing Group UK, (d–f) reproduced with permission [18]. Copyright 2019, Elsevier. (a) The ML framework designed by Lu et al. (b) ML model accuracy comparison with DFT for screened six new HOIPs candidates with suitable bandgap. (c) The final two candidates have direct bandgap and excellent environmental stability. (d) The ML framework designed by Wu et al. (e) Fitting result of DFT bandgap and GBR predicted bandgap. (f) Four representatives newly discovered HOIPs.

One classic ML work on HOIPs was completed by Lu et al. in 2018 [286]. The dataset was collected from previous high‐throughput first‐principles calculations, containing 346 HOIP samples. From these, 212 HOIP compounds with orthorhombic crystal structures and bandgaps calculated using the PBE functional were selected for training; 212 compounds were then divided into a training dataset (80%) and a test dataset (20%). Feature engineering involves selecting and evaluating the initial 30 features (such as ionic radii, tolerance factor, and electronegativity) to describe HOIPs in the chemical space. The GBR algorithm is used for feature selection, employing a “last‐place elimination” procedure to exclude features with less impact on the bandgap, ultimately selecting 14 most important features. In ML training and evaluation stage, six algorithms were chosen under five‐fold cross‐validation, which are GBR, SVR, KRR, GP, DT, and Multilayer Perceptron Regression (MLPR), and evaluating their performance using metrics including the R^2^, r, and MSE. Among these, GBR was found to be the best‐performing model. Model validation involves predicting the bandgaps of all 5504 possible HOIP structures using the trained ML model, initially screening 1669 HOIPs with appropriate bandgaps and further narrowing down to 218 HOIPs suitable for PV applications. Further DFT calculations were conducted to validate the electronic structure, thermal stability, and environmental stability of the selected HOIPs. As shown in Figure 13a, the workflow involves feature engineering, statistical inference, and ML‐based screening to predict potential HOIP candidates, followed by DFT‐based validation for global optimization, bandgap calculation, and stability assessments. The predicted bandgaps of six newly identified lead‐free HOIPs are illustrated in Figure 13b, where a comparison between ML predictions and PBE‐calculated values is provided. These materials exhibit suitable bandgaps for solar cells, making them promising candidates. Additionally, the crystal structures of two screened‐out HOIPs are visualized in Figure 13c, highlighting their octahedral coordination and molecular configurations.

In 2019, Wu et al. [18] used the same target‐driven method as Lu et al. [286] and successfully selected 132 nontoxic and stable candidates from 38230 potential candidates. Notably, unlike the traditional 4:1 train‐test split ratio, Wu et al. used a 90% training and 10% testing split. This split allows the model to better capture the complex features of the data, thereby improving its predictive capability. Additionally, having more training data makes the model more stable and less susceptible to being affected by individual outliers, enhancing its robustness; the potential generalization issues caused by the smaller 10% testing set are addressed through the application of ten‐fold cross‐validation. This method provides a potential solution for future applications of ML in new material discovery, especially when faced with the challenge of insufficient datasets. Figure 13d illustrates the screening process, where charge neutrality and stability filtering reduce the initial dataset to 38086 candidates. ML models (GBR, SVR, KRR) further refine the selection before DFT validation. Figure 13e shows a strong correlation (R^2^ = 0.827) between ML‐predicted and DFT‐calculated bandgaps, confirming model accuracy. Figure 13f presents the crystal structures of selected non‐toxic HOIPs with suitable bandgaps for solar cell applications.

### 9.3 Double Perovskites

**FIGURE 14:**
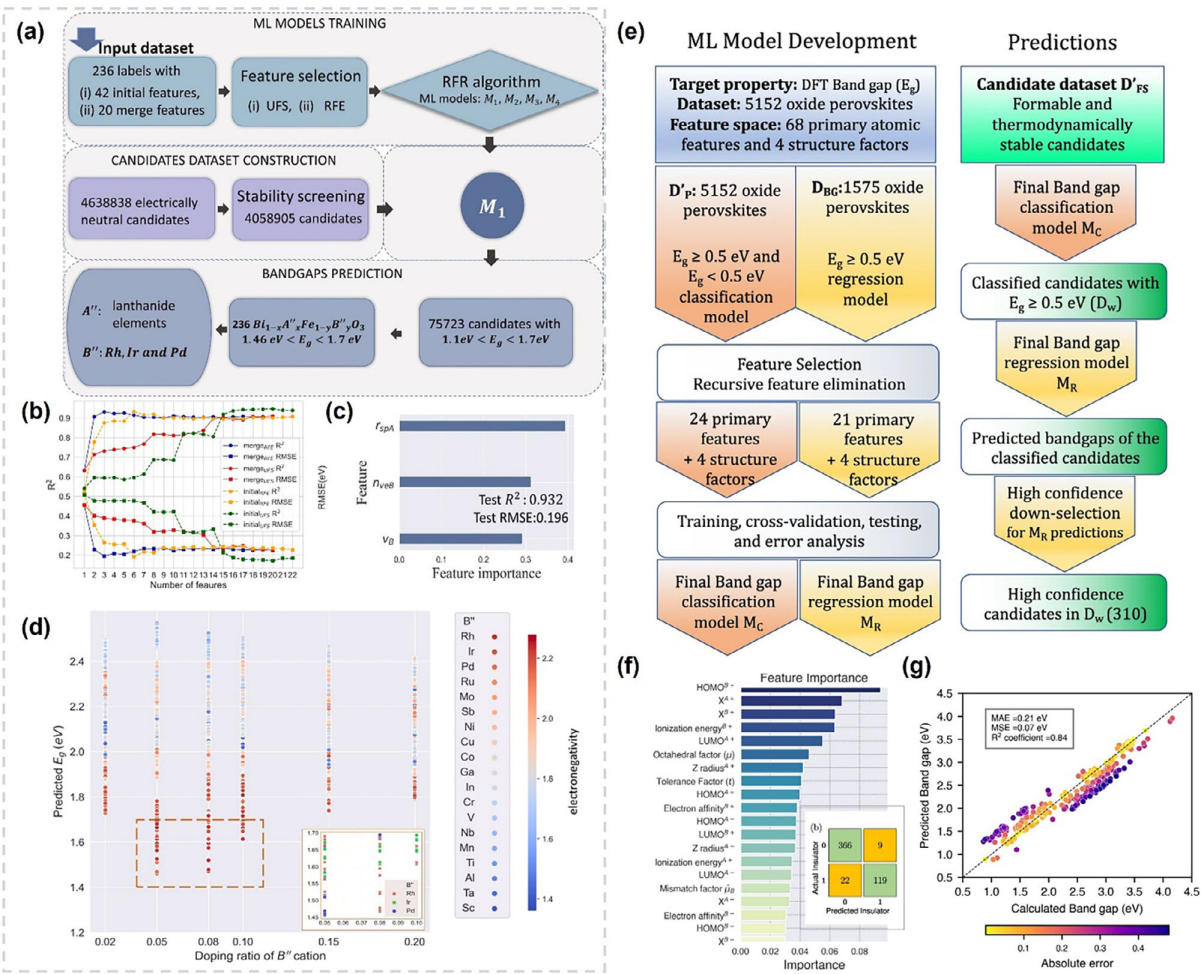
Bandgap prediction of doped double perovskites (a–d) reproduced with permission [287]. Copyright 2022, Elsevier, and application of multi‐model for both classification and prediction of double perovskites (e–g) reproduced under terms of the CC‐BY license [288]. Copyright 2023, The Authors, published by Springer Nature. (a) ML framework designed by Liu et al. for predicting the bandgap of doped double perovskites. (b) Effects of different feature sets and feature selection methods on the prediction performance (R^2^ and RMSE) of the random forest regression model. (c) Feature importance and test set prediction performance metrics for the best RF model (M1). (d) Bandgap prediction distribution of 9576 BiFeO_3_ based double perovskites using model M1, where the color indicates the electronegativity of the doped B″‐site element (the inset shows the specific distribution of doped elements in the 236 best candidate materials with bandgaps between 1.46–1.7 eV). (e) ML framework designed by Talapatra et al. (f) Feature importance analysis. (g) Comparison of actual and calculated bandgap predictions.

In 2022, Liu et al. [287] collected bandgap data for 236 double perovskites from approximately 60 peer‐reviewed publications, including 62 A‐site doped perovskites, 110 B‐site doped perovskites, and 43 A‐site doped perovskites, B‐site co‐doped perovskites and 21 pure perovskites and used 80% of the data set for training and 20% for testing. The feature engineering stage selected 42 initial features related to the electronic structure, the relative position of elements in the periodic table, and their physical properties. The importance of the features is evaluated by calculating the Pearson correlation coefficient between each feature and the bandgap value, and a weighted average of the features according to the A and B positions ensures uniformity. The bandgap value of double perovskite oxide was predicted using RFR, feature selection was done using Recursive Feature Elimination (RFE) and Univariate Feature Selection (UFS), and the optimum feature combination was used to develop the model. The model's performance was assessed using RMSE and R^2^. The finally selected model had an R^2^ value of 0.932 and an RMSE of 0.196 eV on the test set, showing high prediction accuracy. The research team then manually generated the A′_1‐x_A′′_x_B′_1‐y_B′′_y_O_3_ candidate data set and finally obtained 4058905 stable DP oxide materials by screening stability and electroneutrality and applied training. The good model predicted the bandgap values of these materials and screened out 75 723 materials with bandgaps between 1.1 and 1.7 eV, suitable for PV applications. Finally, the model was verified using a validation set that was independent of the training set. The results showed that the bandgap values predicted by the model were in good agreement with the experimental values, verifying the high accuracy and reliability of the model. Figure 14a outlines the ML workflow, from dataset preparation and feature selection to stability screening and bandgap prediction, identifying 75 723 promising candidates. Figure 14b demonstrates the effects of different feature sets and feature selection methods on the prediction performance (R^2^ and RMSE) of the random forest regression model. Figure 14c highlights the feature importance and test set prediction performance metrics for the best random forest model (M1), identifying rspA, nveB, and v_β_ as key influential features. Figure 14d presents the bandgap prediction distribution of 9576 BiFeO_3_‐based double perovskites using model M1, with color intensity indicating the electronegativity of the doped B″‐site element; the inset specifically illustrates the distribution of doped elements among the 236 best candidate materials with bandgaps between 1.46 and 1.7 eV, highlighting Rh, Pd, and Ir as impactful dopants. In 2023, Talapatra et al. [288] construct a multi‐model task for discovering ideal double perovskite. They compiled a dataset of bandgap values for over 5000 materials sourced from the Materials Project database, calculating their bandgaps using DFT. The materials were categorized as either narrow bandgap (<0.5 eV) or wide bandgap (≥0.5 eV). Corresponding descriptors were generated from the dataset, which, combined with the bandgap data, facilitated the construction of two distinct ML models: a binary classification model distinguishing narrow from wide bandgap materials, and a regression model predicting specific bandgap values for materials identified as wide‐bandgap candidates. Hyperparameter tuning was performed using ten‐fold cross‐validation and RandomizedSearchCV, significantly enhancing the predictive accuracy and generalization of the ML models. Model performance metrics included the AUC‐ROC, accuracy, precision, recall, and F1‐score, with the XGBoost algorithm achieving optimal performance and an accuracy of 95% on the test dataset. The optimized models were subsequently applied to an extensive chemical space comprising 68 elements that could potentially form thermodynamically stable double perovskites. This process systematically screened for wide‐bandgap candidate materials, resulting in the identification of 13 589 cubic oxide perovskite compounds. Among these, 310 were predicted with more than 90% confidence in their stability and formability, marking them as promising candidates for further investigation. To provide deeper insights into the ML models' predictions, SHAP analysis was employed. This analysis highlighted the electronegativity range as a critical feature influencing bandgap predictions, revealing that a larger electronegativity range correlates with an increased likelihood of narrow‐bandgap perovskites. Notably, this methodology mirrors the approach previously employed by Li et al. [283], confirming the robustness and replicability of this ML workflow across different inorganic perovskites or PIMs. Additionally, the re‐verification of electronegativity as a critical determinant underscores its broader relevance in predicting perovskite bandgaps. Figure 14e summarizes the ML model development process, outlining steps from feature selection and model validation to the identification of high‐confidence oxide perovskite candidates. Figure 14f details the feature importance analysis, emphasizing key electronic and structural parameters influencing the bandgap classification, alongside the confusion matrix assessing classification performance. Figure 14g presents a comparative analysis of predicted versus DFT‐calculated bandgap values, demonstrating a strong correlation (R^2^ = 0.84) and low MAE = 0.21 eV, reinforcing the reliability and accuracy of the ML model predictions.

### 9.4 Chalcogenide Perovskites

In 2023, Sharma et al. [289] used ML and successfully identified that Ca doping at the Ba site is superior to Ti‐doping at the Zr site. The goal of this research was to identify the optimal dopant to adjust the bandgap of BaZrS_3_ to the optimal range for high‐efficiency PV devices. Since BaZrS_3_ has a direct bandgap of 1.7–1.8 eV, which exceeds the optimal value for single‐junction solar cells (about 1.3 eV), doping is required to reduce the bandgap. Through DFT calculations, a database was created containing 35 different dopants with doping concentrations of 8.33%, 12.5%, and 25%, and calculated the defect formation energies of different dopants to evaluate the stability of the doped structure. In the feature engineering stage, some important chemical descriptors were selected from the Mendeleev database, such as atomic radius, the heat of formation, density, electron affinity, Pauling electronegativity, Glawe number, covalent radius, and dipole polarizability, etc. These descriptors represent various chemical and physical properties of elements. At the same time, to apply these chemical descriptors to the doped BaZrS_3_ structure, a weighted average of the chemical descriptors of each element was performed. The weighted average is obtained by multiplying each descriptor by the number of corresponding elements and dividing its sum by the total number of elements. For example, for BaZrS_3_ doped with calcium and titanium, the weighted average of the chemical descriptors is calculated as follows:

(24) $$\eta_{weighted}=\frac{\sum_{i}\left(x_{i}*\eta_{i}\right)}{N}$$

**FIGURE 15:**
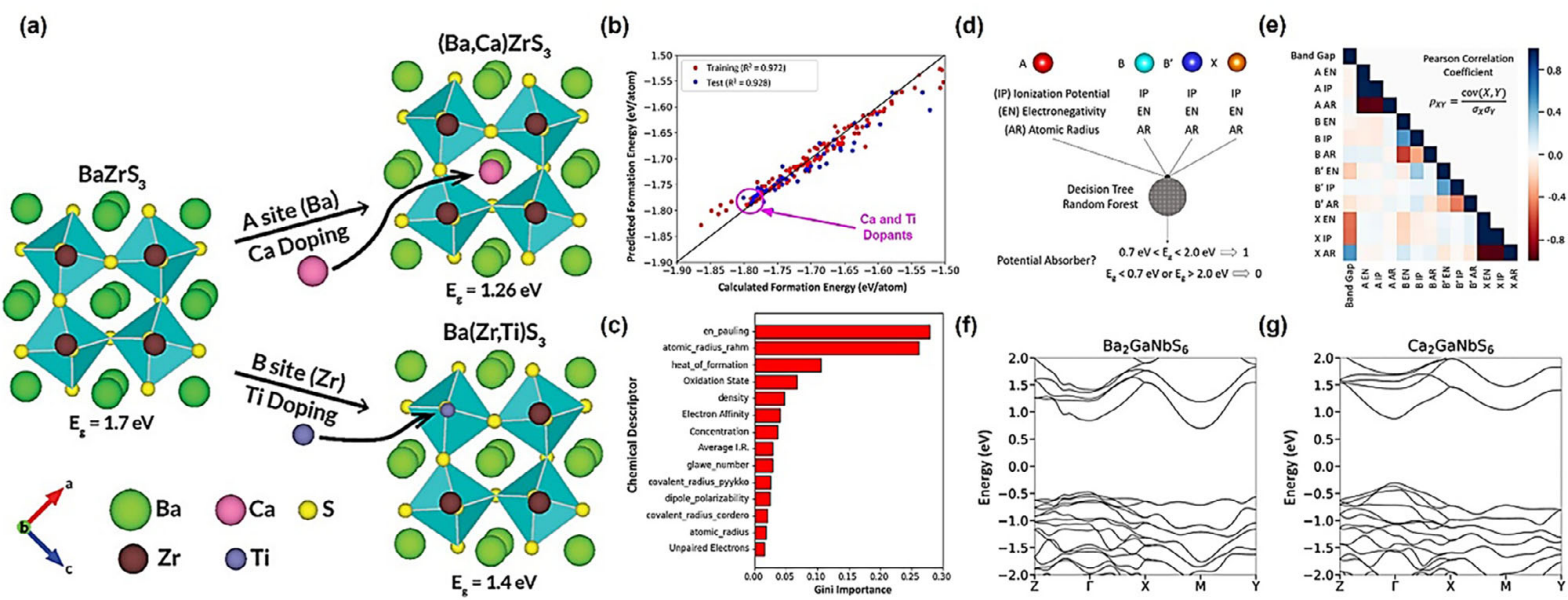
Applications of ML on chalcogenide perovskites (a–c) reproduced with permission [289]. Copyright 2023, American Chemical Society, (d–g) reproduced with permission [290]. Copyright 2019, John Wiley and Sons. (a) Schematic diagram of doping of BaZrS3 in Sharma et al.’s work. (b) Comparison of predicted bandgap and HSE shifted bandgap. (c) Feature importance analysis chart. (d) Workflow of ML designed by Agiorgousis et al. (e) Pearson correlation matrix among 12 input features and bandgap. (f) and (g) Two ideal double chalcogenide perovskites for potential PV application.

Where *x_i_* is the number of elements, i, η_*i*_ is the chemical descriptor of element i, and N is the total number of elements. RFR and Crystal Graph Convolutional Neural Network (CGCNN) were used. RFR was used to predict the bandgap and formation energy and calculated feature importance through the Gini index; CGCNN is based on the deep learning method of graph theory to encode the atomic attributes and relationships in the crystal structure. The data set was divided into a training set (75%) and a test set (25%), and model performance was evaluated by mean absolute error (MAE) and correlation coefficient (R^2^). The MAE of the bandgap prediction is 0.14 eV, the R^2^ of the training set is 0.964, and the R^2^ of the test set is 0.762; the MAE of the formation energy prediction is 0.02 eV/atom, the R^2^ of the training set is 0.971, and the R^2^ of the test set is 0.797. Ca in the A‐site (Ba) and Ti in the B‐site (Zr) was identified as the best dopants by filtering criteria based on bandgap within the Shockley‐Queisser limit (1–1.5 eV) and structural stability. Experimental validation confirmed the accuracy of the theoretical forecast by producing Ca‐doped BaZrS_3_ thin films and conducting measurements on their photoluminescence and bandgaps. Ca‐doped BaZrS_3_ films were synthesized using chemical vapor deposition and the success of doping and structural integrity was confirmed using X‐ray diffraction, scanning electron microscopy, and X‐ray photoelectron spectroscopy. Experimental results showed that Ca doping significantly reduced the bandgap of BaZrS_3_ from about 1.75 to about 1.26 eV, and the doping concentration is lower than 2 at%. In terms of bandgap tuning, Ca doping at the Ba site is better than Ti doping at the Zr site. This study demonstrates the great potential of ML technology in accelerating PV material discovery and optimization. Figure 15a illustrates the effect of A‐site (Ba) Ca doping and B‐site (Zr) Ti doping on the bandgap of BaZrS_3_, reducing it from 1.7 to 1.26 eV and 1.4 eV, respectively. Figure 15b shows the ML‐predicted vs. HSE‐shifted bandgaps, with Ca and Ti doping highlighted near the Shockley‐Queisser limit. Figure 15c presents feature importance analysis, where heat of formation and electronegativity (Pauling scale) are the most influential descriptors in bandgap prediction.

Moreover, Agiorgousis et al. [290] applied ML on the discovery of double chalcogenide perovskites for PV application. In their work, DFT was first used to calculate the structural and electronic properties of 220 initial compounds to ensure that these compounds had stable oxidation states. In the feature engineering stage, the atomic properties, including ionization potential, Pauling electronegativity, and atomic radius in the periodic table, are used as input features. In this work, there are two ML model selection stages. For the first time, the researchers used SVM, RFR, and KRR to directly predict the material's bandgap; each of them conducted 200 runs of training and testing under a ratio of 8:2 on input datasets, and the best hyperparameters of each model were selected through five‐fold cross‐validation. However, the performance of each model on different training set and test set splits is quite different; the test error and standard deviation for each model was SVM: 0.457 ± 0.28 eV, KRR: 0.514 ± 0.294 eV, and RFR 0.466 ± 0.226 eV. Since the predictions of these regression models were not stable enough, classification methods were applied. Due to the poor performance in regression work, KRR was removed, and only retained SVM and RF in the classification work. Classification of materials into potential PV absorbers (bandgap between 0.7 and 2.0 eV) and non‐potential absorbers (bandgap outside this range), the result showed the average accuracy of Random Forest Classifier (RFC) in five‐fold cross‐validation is 86.4%, which outperforms SVM. The trained SVM model was then applied to the entire compound space to screen out more than 450 materials with potential PV properties, which were further evaluated in detail for optical absorption, thermodynamic stability, and kinetic stability. By calculating the dielectric constant, formation energy, etc. of the materials, Ba_2_AlNbS_6_, Ba_2_GaNbS_6_, Ca_2_GaNbS_6_, Sr_2_InNbS_6_, and Ba_2_SnHfS_6_ were identified that have optimal bandgaps and significant optical absorption properties and are also thermodynamically and kinetically stable. Finally, through band structure calculations, it was found that these materials have nearly degenerate indirect and direct bandgaps, as well as low carrier effective masses, making them suitable as efficient PV absorbers. In particular, Ba_2_AlNbS_6_ and Ba_2_GaNbS_6_ have excellent performance in bandgap value and absorption performance and are the most promising solar energy absorption materials. There are many points worth learning and discussing in this work. For example, when the regression task cannot achieve the expected results, the classification task can be used as an alternative. Moreover, the researchers also found that after removing the low correlation features of A‐site cations, the prediction performance of the model was improved. The test errors of RFC and SVM after removing the features of A‐site cations were reduced to 13.28% and 16.70%, respectively, while including the A‐site. The test errors for cationic characteristics are 13.80% and 31.63%, respectively. It can be found that this result is different from the previous example, as discussed in the inorganic perovskite section about Li et al.’s work, which removed high correlation features to optimize model performance. This work removed low‐correlation features and obtained the model's performance optimization. The main reason for this difference is due to the difference in data sets and number of features. When processing a large number of feature sets, high correlation features need to be removed, while for this work, when there are only 12 feature sets, low correlation features need to be removed to remove noise and reduce dimensionality [291, 292]. And why in this work, the A‐site cation has a relatively small effect on the bandgap, which varies in the range of 0.05–0.3 eV. This is different from previous reports that the A‐site electronegativity can effectively affect the bandgap; whether this difference is due to the double chalcogenide perovskite structure is still unknown. Figure 15d presents a DT and RF model utilizing ionization potential, electronegativity, and atomic radius to classify potential absorbers based on bandgap range. Figure 15e shows the Pearson correlation matrix, indicating the relationships between bandgap and elemental descriptors. Figure 15f,g displays the electronic band structures of Ba_2_GaNbS_6_ and Ba_2_AlNbS_6_, respectively, highlighting their band dispersion and potential suitability as absorbers.

### 9.5 Chalcohalide Materials

**FIGURE 16:**
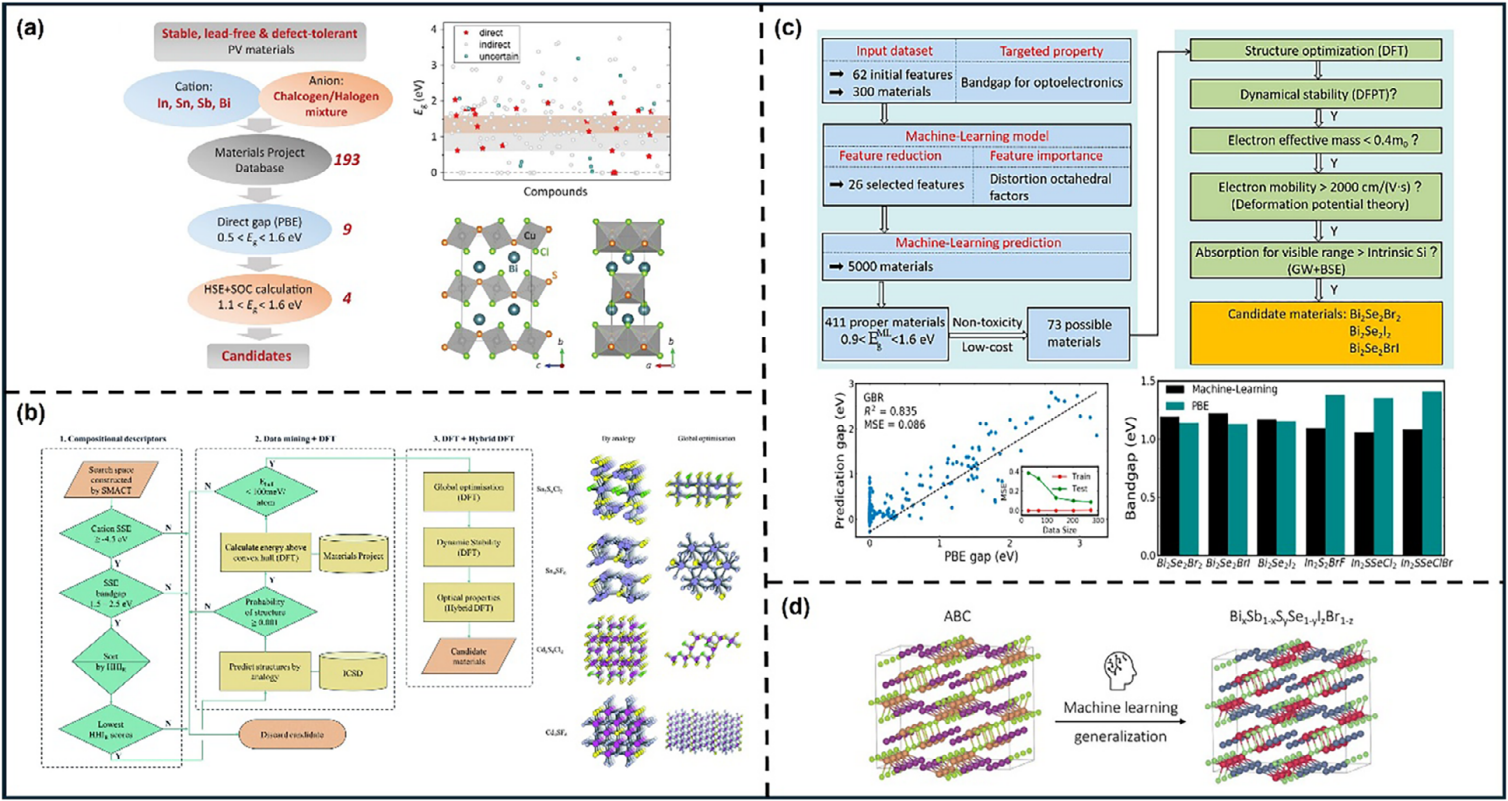
Applications of DFT and ML on chalcohalide materials. (a) High‐throughput screening refined by hybrid functional (HSE+SOC) calculations reproduced with permission [293]. Copyright 2022 John Wiley and Sons. (b) Computer‐aided‐design workflow for chalcohalide structure identification reproduced under terms of the CC‐BY license [294]. Copyright 2018, The Authors, published by Royal Society of Chemistry. (c) ML framework followed by multiple screening criteria including stability, electronic properties, and optical properties reproduced with permission [295]. Copyright 2019, American Chemical Society. (d) CNNs for predicting stability, bandgaps, and optical absorption coefficients of pnictogen chalcohalides reproduced under terms of the CC‐BY license [296]. Copyright 2024, The Authors, published by John Wiley and Sons.

In 2022, Ming et al. [293] utilized DFT computational screening coupled with experimental synthesis to identify stable, lead‐free, defect‐tolerant chalcohalide materials. Their investigation pinpointed CuBiSCl_2_ as a promising candidate with an optimal bandgap of approximately 1.37 eV, validated by comprehensive electronic structure and defect analyses. Although their work did not incorporate ML, their detailed computational screening workflow provides valuable insights applicable to future ML‐driven studies. Specifically, they noted that due to the well‐known underestimation of bandgaps by PBE calculations, broadening the initial bandgap screening range to 0.5–1.6 eV rather than the practical ideal of 1.1–1.6 eV is advisable. Notably, their search space from Material Project, which included cations (In, Sn, Sb, Bi) with lone pairs and large atomic numbers and anions comprising chalcogens (S, Se, Te) and halogens (Cl, Br, I), resulted in only 193 candidates, highlighting significant data shortage compared with perovskites and the pressing need for more extensive computational exploration within PIMs. Figure 16a illustrates the workflow and the structure of the identified compound.

In 2018, Davies et al. [294] proposed a method combining high‐throughput screening and structure prediction ML algorithm aiming to search for new chalcohalide materials for PV applications. The complete workflow and result can be seen in Figure 16b. First, the SMACT library and solid‐state energy (SSE) scale were used to perform preliminary compositional screening of 32 million compounds to ensure that the candidate compounds met the charge neutralization and electronegativity balance requirements and narrowed the candidate compounds to 161 000. Then, the SSE scale was used to screen compounds with a suitable bandgap range (1.5–2.5 eV) to further narrow the candidate range. Next, Pymatgen's structure replacement algorithm was used to predict the crystal structure of the candidate compounds by analogy with known crystal structures. This step provides a preliminary crystal structure model for the candidate compounds. Then, the USPEX evolutionary algorithm was used to perform a global structure search to find the possible lowest energy crystal structure. This stage overcomes the limitations of the analog prediction method and is able to identify new structural types. Through DFT calculations, the researchers evaluated the thermodynamic and kinetic stabilities of these compounds, determining their relative energy positions and their stability relative to phase transitions and decomposition. Four new unreported metal halide sulfide compounds (Sn_5_S_4_Cl_2_, Sn_4_SF_6_, Cd_5_S_4_Cl_2_, and Cd_4_SF_6_) were identified. Despite their slight metastability at 0 K (energy deviations within 100 meV/atom from the convex hull), these materials exhibited significant synthetic feasibility.

In 2019, Ma et al. [295] combined DFT and ML to accelerate the discovery of 2D chalcohalide materials with excellent optoelectronic properties as can be seen in Figure 16c. First, DFT was used to calculate the geometric and electronic properties of 300 2D chalcohalides as datasets. Four different algorithms were trained and evaluated, which are SVR, RFR, Bagging, and GBR. The GBR model performed best in the evaluation of ten‐fold cross‐validation with the lowest mean square error (MSE = 0.086) and the largest coefficient of determination (R^2^ = 0.835). This GBR model was then used to predict the electronic properties of 5000 potential 2D chalcohalide, 411 compounds were screened based on criteria including bandgap, toxicity, cost and kinetic stability. From the 411 materials screened, high‐cost materials were further excluded, and 73 candidate materials were finally selected. These materials were verified by detailed DFT calculations, including evaluation of kinetic stability, effective carrier mass, and carrier mobility. Among the 73 materials, six stable materials with appropriate bandgaps and high carrier mobility were identified. In particular, Bi_2_Se_2_Br_2_, Bi_2_Se_2_BrI, and Bi_2_Se_2_I_2_ showed excellent light absorption ability and optoelectronic properties, making them very suitable for PV applications. This study also introduced the concept of distorted stacking octahedron factor (DSOf) as an improved structural descriptor, which significantly improved the prediction accuracy of ML models. This approach not only accelerates the material discovery process but also provides a general framework for exploring other complex material systems. López et al. [296] employed an integrated bottom‐up approach combining ML models with DFT calculations to predict and optimize the properties of pnictogen chalcohalide for use in PV applications as can be seen in Figure 16d. They constructed a comprehensive dataset by performing DFT calculations on 125 different compositions of pnictogen chalcohalides with general ABC formula (where A = Bi, Sb; B = S, Se; and C = I, Br), focusing on key properties such as thermodynamic stability, energy bandgaps, and optical absorption coefficients. CNNs were used to predict these properties across a much larger compositional space, generating predictions for 9,261 possible candidates. Feature engineering was conducted by using the stoichiometry of the materials as the primary input features, and the model performance was evaluated using MAE through 20‐fold cross‐validation, ensuring robust and reliable results. Although RF models were also tested, they were found to be less accurate compared to CNNs and thus played a secondary role, primarily serving as a benchmark for model comparison. After obtaining the CNNs predictions, they filtered the results to focus on thermodynamically stable compositions, using a threshold of 0.1 eV per atom for the formation enthalpy, below which materials were considered stable against phase segregation. This filtering process narrowed down the candidate materials to those with the most promising properties for PV applications and successfully identified Bi_0.3_Sb_0.7_SeI as the ideal absorber layer.

## 10 Summary of Applications

Here, we summarize the application of ML on different perovskites and PIMs, as shown in Table 2. The table systematically shows all the applications of ML algorithms we mentioned in previous parts, including target prediction, the algorithms used, and their evaluation results.

**TABLE 2:** Summary of ML applications on different materials with used algorithms and evaluation (metrics).

Material type	Target	Algorithms	Evaluation
Inorganic perovskites	lattice constant	SVM, ANN	SVM: PAD < 0.7% on training data, < 1% on testing data
Inorganic halide perovskites	Bandgap classification	XGBoost, SVC, MLP, RF	XGBoost: Precision 95%, Recall 78%, F1 Score 0.86, AUC 0.89
HOIPs	Bandgap prediction	GBR, SVR, KRR, GPR, DTR, MLPR	GBR: R^2^ = 0.97, Pearson's r = 0.985, MSE = 0.085
HOIPs	Bandgap prediction	GBR, SVR, KRR	GBR: R^2^ = 0.827, MAE = 0.377, MSE = 0.201
Double perovskites	Bandgap prediction	RFR	R^2^ = 0.932, RMSE = 0.196 eV
Double perovskites	Bandgap classification and regression	RFR, RFC	Classification AUC = 0.98, Regression MAE = 0.18 eV, R^2^ = 0.86
Chalcogenide perovskite	Bandgap prediction and formation energy	RFR	MAE = 0.14 eV for bandgap prediction, MAE = 0.02 eV for formation energy
Chalcogenide double perovskites	Bandgap prediction	SVM, RFR, KRR (regression); SVM, RFC (classification)	Regression: SVM MAE = 0.457 eV, RFR MAE = 0.466 eV; Classification: RFC accuracy = 86.4%
Chalcohalides	Bandgap and stability prediction	Pymatgen (structure prediction), USPEX (global structure search), DFT	Energy deviation < 100 meV/atom, Bandgap: 0.9‐2.75 eV
2D octahedral chalcohalides	Bandgap and carrier mobility prediction	GBR, SVR, RFR, Bagging	GBR: R^2^ = 0.835, MSE = 0.086
Pnictogen chalcohalides	Bandgap, stability, and optical properties	CNN, RF	MAE for energy and optical properties predictions, Convex hull stability analysis

## 11 Conclusions and Outlook

In conclusion, integrating machine learning into the design, discovery and optimization of non‐toxic and stable polymeric materials, along with other associated material systems, represents a transformative step toward next‐generation solar absorber materials. Rapid advances in computational power and the growing availability of materials data have enabled ML techniques to address persistent challenges in toxicity, stability, and scalability more effectively than traditional trial‐and‐error approaches.

In this review, we presented a comprehensive analysis of ML applications in perovskites and PIMs, highlighting shared chemical and structural characteristics, while emphasizing the major challenges, particularly the scarcity of high‐quality datasets for emerging PIMs. To address this, we outlined two complementary strategies: multi‐fidelity learning, which integrates datasets of varying accuracy and cost to improve robustness; and active learning, which accelerates data generation by prioritizing the most informative samples.

One promising strategy for advancing PIMs is the transfer of established ML workflows from halide perovskites. We demonstrated how transferable descriptors, particularly electron affinity, a strong predictor of electronic structure and stability, can enable accurate predication in PIMs with minimal model retraining. We also introduced automated feature engineering tools such as Matminer and discussed how its integration with techniques like RFE and PCA enhances model performance and interpretability.

We also reviewed a range of ML algorithms, from classical models like SVM and RF, to modern techniques such as gradient boost (e.g., XGBoost) and generative models, which enable inverse materials design by proposing novel crystal structures. We further highlight the importance of model interpretability, as means to link ML outputs to physically meaningful insights and broaden accessibility across different disciplines such as materials science, chemistry, device physics, engineering and even among experimentalists and technology developers.

By systematically reviewing the state‐of‐the‐art, identifying unresolved challenges, and proposing practical solutions, this review offers a comprehensive roadmap for the ML‐guided discovery of high‐efficiency, low‐toxicity, and stable solar absorber materials. We anticipate that these insights will further catalyze interdisciplinary collaboration and accelerate the development of commercially viable, efficient, and sustainable solar conversion technologies.

## Conflicts of Interest

The authors declare no conflict of interest.

## Supporting information




**Supporting file**: advs74952‐sup‐0001‐SuppMat.docx

## Data Availability

Data sharing not applicable to this article as no datasets were generated or analysed during the current study.
