# Supplementary material for: Machine Learning for Designing Perovskites and Perovskite‐Inspired Solar Materials: Emerging Opportunities and Challenges
Source: Adv Sci (Weinh). 2026 Mar 24;13(23):e74952. doi: 10.1002/advs.74952 (PMC13104142; doi:10.1002/advs.74952)
Supplement: Supplementary file 1 — Supporting file: advs74952‐sup‐0001‐SuppMat.docx [file ADVS-13-e74952-s001.docx]

Supplementary Information

**Machine Learning for Designing Perovskites and Perovskite-inspired Solar Materials: Emerging Opportunities and Challenges**

*Yangfan Zhang, Yiming Xia, Ali Shakiba, Hongrui Zhang, Xiaojing Hao, ^*^ Priyank V. Kumar, ^*^ Mahesh P. Suryawanshi^*^*

*Yangfan Zhang, Yiming Xia, Ali Shakiba, Hongrui Zhang, Xiaojing Hao, Mahesh P. Suryawanshi*

School of Photovoltaic and Renewable Energy Engineering, University of New South Wales, Sydney, New South Wales, 2052, Australia

Email: m.suryawanshi@unsw.edu.au (Mahesh P. Suryawanshi)

*Priyank V. Kumar*

School of Chemical Engineering, University of New South Wales, Sydney, New South Wales, 2052, Australia.

**Supplementary Figures**


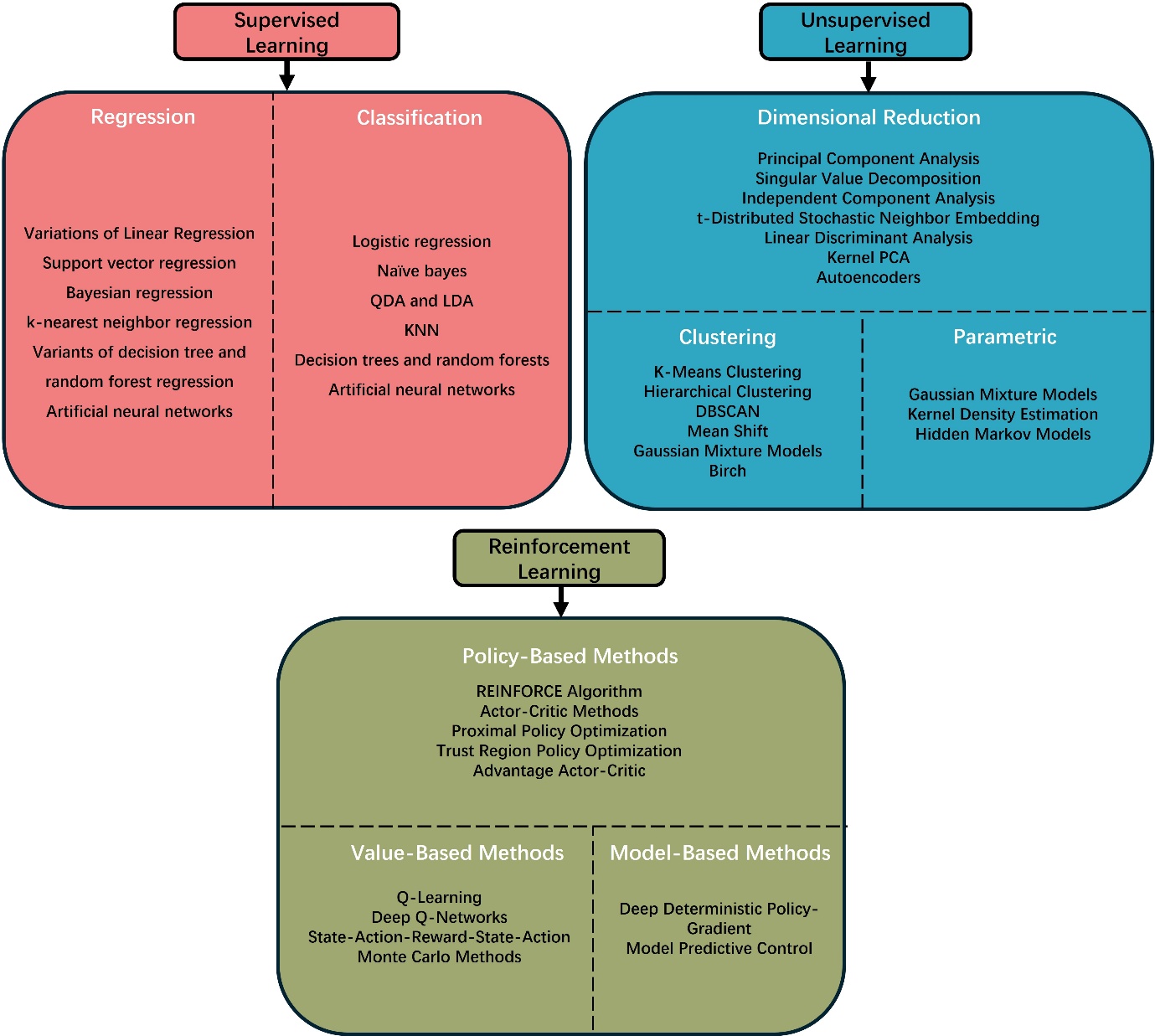


**Supplementary Figure 1.** An overview of three categories of ML: supervised learning, unsupervised learning, and reinforcement learning, along with some of the algorithms belonging to each category.


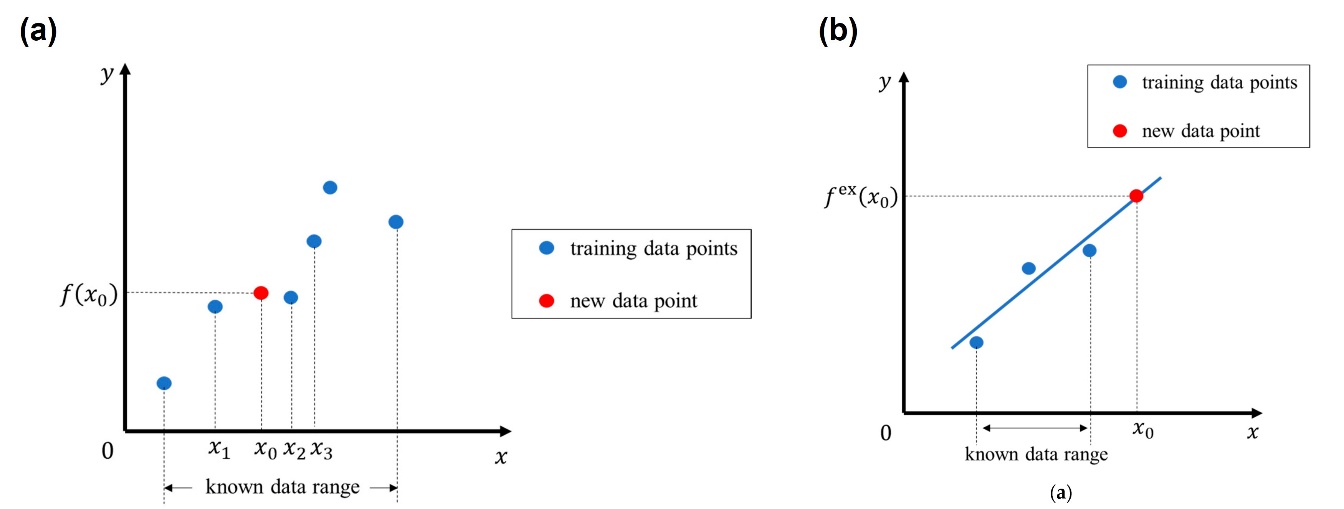


**Supplementary Figure 2.** Illustration of interpolation and extrapolation from ref.[1] (a) Interpolation of data within the given data range. (b) Extrapolation of data exceeds the given data range.

**Supplementary Tables**

**Supplementary Table 1** Abbreviations and explanation.

| **Field** | **Abbreviation** | **Full name** |
| --- | --- | --- |
| **Material related** | PIMs | Perovskite-inspired materials |
|  | ML | Machine learning |
|  | PV | photovoltaic |
|  | LHPs | Lead (Pb)-based halide perovskites |
|  | PCE(s) | power conversion efficiencies |
|  | HOIPs | hybrid organic-inorganic perovskites |
|  | DFT | density functional theory |
|  | MLIPs | machine-learned interatomic potentials |
|  | PC | photocatalysis |
|  | RP | Ruddlesden–Popper |
|  | DJ | Dion–Jacobson |
|  | ACI | alternating cation interlayer |
|  | QW | Quantum Well |
|  | CBM | Conduction Band Minimum |
|  | VB | Valence Band |
|  | Eb | Exciton Binding Energy |
|  | SQ | Shockley–Queisser |
|  | Ef | Formation Energy |
|  | Ehull | Energy Above Convex Hull |
|  | BA | Butylammonium |
|  | PEA | Phenylethylammonium |
|  | FA | Formamidinium |
|  | MA | Methylammonium |
|  | BEA | Butanediamine |
|  | AMP | Aminomethyl Piperidinium |
|  | SLME | Spectroscopically Limited Maximum Efficiency |
| **Machine learning related** | ML | Machine Learning |
|  | DFT | Density Functional Theory |
|  | GW | Green’s Function and Screened Coulomb Interaction Method |
|  | AIMD | Ab Initio Molecular Dynamics |
|  | MLIP | Machine-Learned Interatomic Potential |
|  | MP | Materials Project |
|  | ICSD | Inorganic Crystal Structure Database |
|  | OQMD | Open Quantum Materials Database |
|  | PU | Positive-Unlabelled (Learning) |
|  | CLscore | Crystal-Likeness Score |
|  | CSLLM | Crystal Synthesis Large Language Model |
|  | UQ | Uncertainty Quantification |
|  | SVM | Support Vector Machine |
|  | KRR | Kernel Ridge Regression |
|  | RF | Random Forest |
|  | XGBoost | eXtreme Gradient Boosting |
|  | LightGBM | Light Gradient Boosting Machine |
|  | MLP | Multilayer Perceptron |
|  | ANN | Artificial Neural Network |
|  | CNN | Convolutional Neural Network |
|  | GNN | Graph Neural Network |
|  | GBDT | Gradient Boosted Decision Tree |
|  | SFS | Sequential Forward Selection |
|  | PCA | Principal Component Analysis |
|  | SHAP | SHapley Additive exPlanations |
|  | RFE | Recursive Feature Elimination |
|  | LR | Linear Regression |
|  | SVR | Support Vector Regression |
|  | KNN | K-Nearest Neighbour |
|  | RMSE | Root Mean Square Error |
|  | R² | Coefficient of Determination |
|  | MAE | Mean Absolute Error |

**Supplementary Table 2** High-throughput screening tools

| Name | Function | URL |
| --- | --- | --- |
| AFLOW *π* | A framework for automating first principles calculations in materials discovery. | http://aflowlib.org/src/aflowpi |
| AiiDA | Infrastructure for automating, managing, and storing computational science workflows and data. | http://www.aiida.net |
| ASE | A package for setting up, running, and analyzing atomistic simulations. | https://wiki.fysik.dtu.dk/ase |
| Atomate | A Python library for automating materials science simulations with pre-built workflows. | https://atomate.org |
| FireWorks | Workflow software for automating the execution of computational tasks and simulations. | https://materialsproject.github.io/fireworks |
| MPInterfaces | A toolkit for the analysis of material interfaces, integrating with various simulation tools. | http://henniggroup.github.io/MPInterfaces |
| Pymatflow | A tool to simplify the execution of ab initio simulations in materials science. | http://pymatflow.readthedocs.org |
| Pylada | A Python framework for orchestrating physics simulations, offering customization for specific tasks. | http://pylada.github.io/pylada |
| Pyiron | An integrated development environment for computational materials science, integrating simulation management and analysis. | https://pyiron.github.io |
| Pymatgen | A Python library for materials analysis, offering tools for manipulation of crystal structures and electronic band structures. | https://pymatgen.org |

**Supplementary Table 3** Key geometric and electronic features for perovskites and PIMs from ref.[2]

| Features | Property description |
| --- | --- |
| $r_{A},r_{B} and r_{X}$ | Ionic radii for the A-, B-, and X-site atoms |
| $T_{f}$ | Tolerance factor defined as $\frac{r_{A}^{i}+r_{x}^{i}}{\sqrt{2}(r_{B}^{i}+r_{x}^{i})}$ |
| $O_{f}$ | Octahedral factor defined as $\frac{r_{B}^{i}}{r_{x}^{i}}$ |
| $\chi_{B},\chi_{X}$ | Martynov-Batsanov electronegativity scales |
| $r_{A}^{s+p},r_{B}^{s+p},r_{X}^{s+p}$ | Sums of the s and p orbital radii for the A, B, and X atoms |
| $r_{A-X}^{b},r_{B-X}^{b}$ | Bond valence radii for A–X and B–X bonds |
| $A_{x-electron}$(x=s,p,d,f)  $B_{x-electron}$(x=s,p,d,f)  $X_{x-electron}$(x=s,p,d,f) | Numbers of s, p, d and f orbital electron for the A-, B- and X-site |
| $P_{A}, P_{B}, P_{X}$ | Ionic polarizability for the A-, B- and X-site ionic |
| $\Delta\chi_{A-X}^{MB}, \Delta\chi_{B-X}^{MB}$ | Differences in the Martynov–Batsanov electronegativity scales for A–X and B–X atoms |
| ${HOMO}_{A},{LUMO}_{A}$ | HOMO and LUMO for the A-site molecules (HOIPs only) |
| ${IE}_{B}$ | Ionization energy for the B-site cations |
| ${EA}_{B}$ | Electron affinity for the B-site atoms |
| $1^{st}{IP}_{B}, 1^{st}{IP}_{X}$ | The first ionization energy for B- and X-site atoms |
| ${IC}_{A}, {IC}_{B}, {IC}_{X}$ | Ionic charge for A-, B- and X- site |
| ${VE}_{B}$ | Valence electrons for the B-site atoms |

**References**

1. Jiang, B., et al., *Integrating Interpolation and Extrapolation: A Hybrid Predictive Framework for Supervised Learning.* Applied Sciences, 2024. **14**(15): p. 6414.

2. Lu, S., et al., *Accelerated discovery of stable lead-free hybrid organic-inorganic perovskites via machine learning.* Nature communications, 2018. **9**(1): p. 3405.
